# Integration of metabolomics and peptidomics reveals distinct molecular landscape of human diabetic kidney disease

**DOI:** 10.7150/thno.80435

**Published:** 2023-05-21

**Authors:** Xinrong Jiang, Xingyue Liu, Xuetong Qu, Pingya Zhu, Fangjie Wo, Xinran Xu, Juan Jin, Qiang He, Jianmin Wu

**Affiliations:** 1 Institution of Analytical Chemistry, Department of Chemistry, Zhejiang University, Hangzhou, 310058, China.; 2 Department of Nephrology, The First Affiliated Hospital of Zhejiang Chinese Medical University, Zhejiang Provincial Hospital of Traditional Chinese Medicine, Hangzhou, 310006, China.; 3 Department of Nephrology, The First People's Hospital of Hangzhou Lin'an District, Affiliated Lin'an People's Hospital of Hangzhou Medical College, Hangzhou, 311300, China.; 4 Well-healthcare Technologies Co., Hangzhou, 310051, China.

**Keywords:** Multi-omics, Diabetic kidney disease, Type 2 diabetes, Non-invasive diagnosis, Laser desorption/ionization mass spectrometry

## Abstract

Diabetic kidney disease (DKD) is the most common microvascular complication of diabetes, and there is an urgent need to discover reliable biomarkers for early diagnosis. Here, we established an effective urine multi-omics platform and integrated metabolomics and peptidomics to investigate the biological changes during DKD pathogenesis.

**Methods:** Totally 766 volunteers (221 HC, 198 T2DM, 175 early DKD, 125 overt DKD, and 47 grey-zone T2DM patients with abnormal urinary mALB concentration) were included in this study. Non-targeted metabolic fingerprints of urine samples were acquired on matrix-free LDI-MS platform by the tip-contact extraction method using fluorinated ethylene propylene coated silicon nanowires chips (FEP@SiNWs), while peptide profiles hidden in urine samples were uncovered by MALDI-TOF MS after capturing urine peptides by porous silicon microparticles.

**Results:** After multivariate analysis, ten metabolites and six peptides were verified to be stepwise regulated in different DKD stages. The altered metabolic pathways and biological processes associated with the DKD pathogenesis were concentrated in amino acid metabolism and cellular protein metabolic process, which were supported by renal transcriptomics. Interestingly, multi-omics significantly increased the diagnostic accuracy for both early DKD diagnosis and DKD status discrimination. Combined with machine learning, a stepwise prediction model was constructed and 89.9% of HC, 75.5% of T2DM, 69.6% of early DKD and 75.7% of overt DKD subjects in the external validation cohort were correctly classified. In addition, 87.5% of grey-zone patients were successfully distinguished from T2DM patients.

**Conclusion:** This multi-omics platform displayed a satisfactory ability to explore molecular information and provided a new insight for establishing effective DKD management.

## Introduction

Diabetic kidney disease (DKD) is one of the major microvascular complications of diabetes, which has become the leading cause of chronic kidney disease and end-stage renal disease 1,2. In recent years, much efforts have been made to build an effective diabetes mellitus (DM) management system. Nevertheless, it remains a challenge to prevent the incidence of DM-related complications, such as damage to kidneys and other organs 3,4. Urinary albumin-to-creatinine ratio (UACR) and estimated glomerular filtration rate (eGFR) are commonly recognized metrics to monitor DKD status 5,6. However, an increasing body of evidence has suggested that the progression of renal function damage may not accompany the deterioration of proteinuria in diabetes, and early progressive renal function decline may be observed before the onset of microalbuminuria in a considerable proportion of DM patients 5,7,8. A meta-analysis revealed that the misclassification rate of accurate DKD diagnosis reaches 49.2% if based on clinical information alone 9. Thus, there is an urgent need for more sensitive and specific biomarkers that can predict early-stage renal injury and assess the DKD progression. On the other hand, although several factors have been proposed to be involved in the DKD onset such as the increased activity of protein kinase C, the underlying molecular mechanisms are still debated and not well understood 10.

Compared with genomics, peptidomics and metabolomics create a novel window to elucidate intermediate and end products of biological pathways. Driven by the development of high-throughput technologies, peptidomics and metabolomics have been rapidly applied to specific clinical settings 11,12. Recent years, the mining of potential biomarkers for early DKD diagnosis based on peptidomics or metabolomics has been carried out successively 13-15. Notably, a panel consisting of 273 urinary peptides exhibited high accuracy in the cross-sectional classification of eGFR status and predicted rapid progression of eGFR better than albumin excretion rate 16,17. Besides, several metabolites were identified to be associated with kidney function decline, such as aconitic acid, citric acid, and uracil 15. An increasing body of evidence has suggested that a panel composed of multiple biomarkers exhibits great priorities in improving accuracy, enhancing diagnosis, and reducing misclassifications 18,19. Therefore, we hypothesized that integrative omics may provide an unprecedented view of understanding DKD mechanisms and discovering potential biomarkers that enable effective early DKD diagnosis.

In contrast with other methods such as NMR spectroscopy, mass spectrometry (MS) exhibits priorities in detecting hundreds of substances simultaneously without labeling or preselection 20. MALDI-TOF MS has been widely regarded as a high-throughput platform for profiling biological samples, which can meet the requirements for large-scale clinical diagnosis 21,22. To acquire the unique peptide information in biofluids, our group has reported porous silicon-based MALDI-MS technology to capture peptides and exclude abundant proteins in serum samples 23,24. The structural information of differential peptides was obtained by nano-LC-ESI MS/MS analysis. Besides, our previous work has established a tip-contact extraction (TCE) method coupled with LDI-MS platform, which is applicable for ultrasensitive and reproducible detection of urinary metabolites 25,26. For significantly perturbed metabolites, identifications were achieved by matching exact mass and high-resolution MS/MS spectra to databases and standard samples. On the basis of these works, we intend to construct a urine multi-omics platform to identify potential biomarkers associated with DKD progression, understand the pathogenesis mechanism, and achieve non-invasive diagnosis in the population.

## Methods

### Participant recruitment

Participants were recruited consecutively in the diabetes center from Zhejiang Provincial People's Hospital between 2020 and 2022. Volunteers were excluded when renal impairment was likely caused by other diseases such as polycystic kidney disease, presence of overt hematuria, or history of glomerulonephritis. The following groups were defined according to the albuminuria category classified by the Kidney Disease: Improving Global Outcomes (KDIGO) Diabetes Work Group 27: HC with negative urine routine results and no abnormal renal biochemistry; T2DM patients without micro- or macroalbuminuria (mALB < 30 mg/g, UACR < 3 mg/mmol, and eGFR ≥ 60 ml/min per 1.73 m^2^) and abnormal renal biochemistry; early DKD patients with microalbuminuria (3 ≤ UACR < 30 mg/mmol, and eGFR ≥ 60 ml/min per 1.73 m^2^), overt DKD patients with macroalbuminuria and typical diabetic glomerulopathy on renal biopsy (UACR ≥ 30 mg/mmol or eGFR < ml/min per 1.73 m^2^), grey-zone T2DM patients with abnormal urinary microalbumin concentration (mALB ≥ 30 mg/g, UACR < 3 mg/mmol, and eGFR ≥ ml/min per 1.73 m^2^). Volunteers with a history of urinary diseases or other metabolic disorders were excluded in study. Totally, 221 HC, 198 T2DM, 175 early DKD, 125 overt DKD, and 47 grey-zone patients were selected in terms of matched gender, age, body mass index (BMI), and smoking status. The clinical characteristics of all participants are shown in Table S1.

### Sample collection and preparation

Urine specimens were collected at the midstream of first morning urine on an empty stomach at around 7:00 to 9:00 a.m. Cell debris and insoluble residues were removed by centrifugation (8000 g for 10 min at 4 °C) and the final supernatant was stored in a refrigerator at -80 °C until use. Prior to MS analysis, urine samples were thawed at 4 °C. Metabolite extraction was performed by tip-contact sampling process using FEP@SiNWs chips and the detailed extraction procedure has been described in our previous work 25. Briefly, 20 μL of urine samples were dropped onto ITO glass and then a FEP@SiNWs chip (4 mm × 4 mm) was attached to the surface of droplet. After retaining for 20 min, metabolites in urine samples were extracted onto the substrate and the excess urine was removed by N_2_ stream. Effective peptide capture and protease exclusion were achieved by Si microparticles with suitable pore size. In detail, urine samples were diluted with ultrapure water (v/v 1:1) and then added to 96-well plates loaded with porous Si microparticles. After shaking at 1350 rpm for 15 min, the suspension was centrifuged at 2220 g for 2 min and the supernatant was removed from the microparticles. Subsequently, the particles were re-suspended in ultrapure water and isolated by centrifugation at 2220 g for 2 min to eliminate salt interference.

### Urine peptidomic and metabolomic profiling on LDI-MS platform

Metabolic fingerprints of urine samples were recorded by ultrafleXtreme MALDI-TOF/TOF instrument (Bruker Daltonics Co.) equipped with a 355 nm Nd:YAG laser beam. After metabolite extraction, FEP@SiNW chips were stuck onto the custom-made aluminum plate and then inserted into the instrument. The relative laser pulse energy was set at 57% of the total energy to conduct data acquisition in reflecting negative ion mode. The lens was set at 8.50 kV and the voltage of ion source 1, ion source 2, reflector 1 and reflector 2 was set at 20.00, 17.75, 21.10 and 10.70 kV, respectively. The pulsed ion extraction time for urine detection was optimized to 120 ns and the laser parameter was set at 4_large. The m/z range was set at 20-350 Da with 2000 accumulation laser shots per sample. For urine metabolomic profiling, three replicates were performed in all cases.

For peptidomics analysis, the mass spectra of captured peptides were obtained on ClinMS-Plat I MALDI-TOF/TOF mass spectrometer (Well-healthcare Technologies Co.) equipped with a 337 nm nitrogen laser. The microparticles were suspended in a solution of ultrapure water, acetonitrile and TFA (v/v 50:50:0.1) and then spotted onto the MALDI plate. After drying, 1 μL of α-cyano-4-hydroxycinnamic acid (CHCA) dissolved in a mixture of ultrapure water, acetonitrile and TFA (v/v 50:50:0.1) was added to each spot and the samples were air-dried before insertion into the instrument. MS spectra were acquired at an m/z range of 600-20000 Da in linear positive ion mode. The voltage of detector, repeller, extractor and focus lens was set at 2.77, 20.00, 1.95, and 7.00 kV, respectively. The frequency was 60 Hz and the delay time was set at 250 ns. The laser pulse energy was 41.8 μJ and 800 laser shots were averaged for each mass spectrum. For urine peptidomic profiling, three replicates were performed in all cases.

### Identification of the dysregulated metabolites and peptides

The structural identification of differential metabolites was performed by matching the mass spectra with the Human Metabolome Database (HMDB, http://www.hmdb.ca/) and commercial standard reagents. Firstly, UPLC-MS/MS analysis of pooled urine samples provided the exact mass and fragment profile, which were utilized to identify metabolites through database searching. The relative error of the exact m/z value was limited to 30 ppm and a possible list of differential metabolites was acquired. Subsequently, the initially identified metabolites were verified by matching the exact mass and MS/MS spectra from urine samples with the purchased standard reagents on MALDI-TOF/TOF tandem mass spectrometry. The detailed experimental parameters of UPLC-MS analysis were provided in ESI†.

The peptides captured in porous Si microparticles were identified by nano-LC-ESI MS/MS analysis with a 10 kDa ultrafiltration centrifuge tube. The obtained exact mass and MS/MS spectra were searched against the Swiss-Prot database with the open pFind3 search engine, in which no enzyme was selected in the digestion section. The max missing cleavage number was set as 3 and the error margin of exact mass and fragment peaks was set as 20 ppm. Oxidation [M] and oxidation [P] were set as variable modifications and no fixed modification was added. Automated filtering was performed and peptides scoring with q-values (false discovery rate filter) ≤ 0.01 were retained. The detailed experimental parameters of nano-LC-ESI MS/MS analysis were provided in ESI†.

### Statistical analysis

Metabolomics and peptidomics data were processed by FlexAnalysis (Bruker Daltonics Co.) and ClinMS Analyzer (Zhejiang Huijian AIMS Technology Co.), respectively. Peaks with S/N > 3 were picked for subsequent statistical analysis. After normalizing the obtained mass spectra with the cubic spline method in R 3.5.2 software, differential metabolites and peptides were sorted out by student's t-test using MATLAB software and the false discovery rate (FDR) value is calculated by the R function *p.adjust* based on the Benjamini-Hochberg method. To evaluate the molecular distinctions between groups, orthogonal partial least squares discriminant analysis (OPLS-DA) was performed by SIMCA software. To construct an effective diagnostic model, seven machine learning algorithms were established in MATLAB software via cross-validation (10-folds) and externally validated on the held-out 10% test set, including support vector machine (SVM), decision trees (DT), naïve Bayesian classifier (NB), logistic regression (Logi), linear discriminant analysis (LDA), nearest neighbor classifier (KNN), and LASSO regression. Accuracy, F-measure, kappa coefficient and precision were utilized to evaluate the model performances of the different classification methods. Besides, receiver operating curve (ROC) analysis was conducted to measure diagnostic metrics, including the area under the curve (AUC), specificity, and sensitivity. What's more, the classification results for the training and validation datasets were displayed in confusion matrix. The disturbed metabolic pathways were pictured by MetaboAnalyst and intersection analysis of enriched pathways was carried out with TBtools. To highlight the potential functional relationships between dysregulated metabolites, network analysis was performed using MetaboAnalyst. The biological processes involved in the attributed proteins were annotated by GO database and the significantly enriched biological processes for each stage were identified using Biological Networks Gene Ontology. Besides, differentially expressed peptides and metabolites were utilized as input into MetaboAnalyst to perform joint-pathway analysis. What's more, a compound-reaction-enzyme-gene network was built by MetScape to acquire related metabolic enzyme genes and pathways. To investigate the molecular mechanisms during DKD pathogenesis, RNASeq data in glomeruli from human kidneys with DKD and morphologically normal kidneys were downloaded from the publicly available database (GSE1009). The detailed procedure of transcriptome analysis was provided in ESI†.

### Study approval

Urine samples were obtained from Zhejiang Provincial People's Hospital. The Ethical Committee of the Zhejiang Provincial People's Hospital approved the protocol (No. 2021KY020) and the study was performed in accordance with the ethical standards laid down in the 1964 Declaration of Helsinki and its later amendments. Written informed consent from each patient was achieved.

## Results

### Multi-omics Platform and quality control assessment

**Scheme 1:**
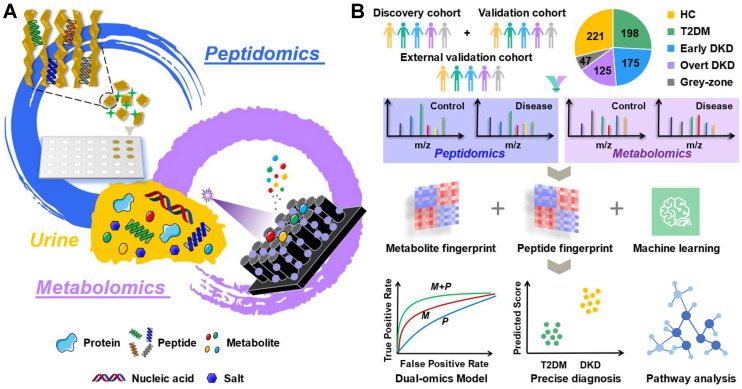
The workflow of urine multi-omics analysis. (A) Mining metabolite and peptide information in urine samples based on porous Si microparticles and FEP@SiNWs chips. (B) Establishing effective DKD diagnostic model and investigating molecular mechanism.

The procedure of the multi-omics analysis of urine samples to identify DKD-related biomarkers is presented in Scheme 1. In this study, non-targeted metabolic profiles of urine samples were acquired by TCE method based on FEP@SiNWs chip coupled with LDI-MS detection 25, while peptide profiles in urine samples was uncovered by MALDI-TOF MS after capturing urine peptides by porous silicon microparticles 23. A pooled quality control (QC) sample was prepared by pooling equal aliquots from all samples and further utilized to ensure the quality of acquired mass spectra. As shown in Figure S1, the metabolic and peptide profiles of the QC samples and healthy controls collected in each independent batch of experiments were clustered together, indicating the reliability of collective data. In addition, we observed acceptable reproducibility for the QC sample in the repeated measurements. As shown in Figure S2, spearman coefficients for metabolite and peptide abundance detected in QC samples from two technical replicates were 0.884 and 0.955, respectively. The medium RSD of intra-batch measurements for metabolites and peptides is 10.17% and 7.55%, respectively. As to the inter-batch stability, the medium RSD for metabolite and peptide is 18.74% and 9.37%, respectively. To check the stability of the obtained metabolic fingerprints, the peak ratios of two internal standards (2-chlorophenylalanine, ketoprofen) spiked in urine samples were evaluated. Figure S3 displayed that the ratios of two internal standard behaved quite stable in each independent measurement, with the RSD being less than 25%. Notably, we have reviewed current guidelines of the US Food Drug Administration with respect to requirements for defining assay performance and provided standard curve, accuracy, precision, recovery, stability (extraction stability, freeze-thaw stability, and long-term stability), dilution effect, and repeatability within and between batches as required (Figure S4-S13). Furthermore, data-driven normalization for urine samples have been screened according to the methods proposed in our previous work 25. The cubic spline normalization method was chosen for subsequent analysis. These results gave us confidence that the established multi-omics platform has robust performance for acquiring reproducible MS profiles.

### Metabolomic landscape and peptide pattern of DKD and controls

**Figure 1:**
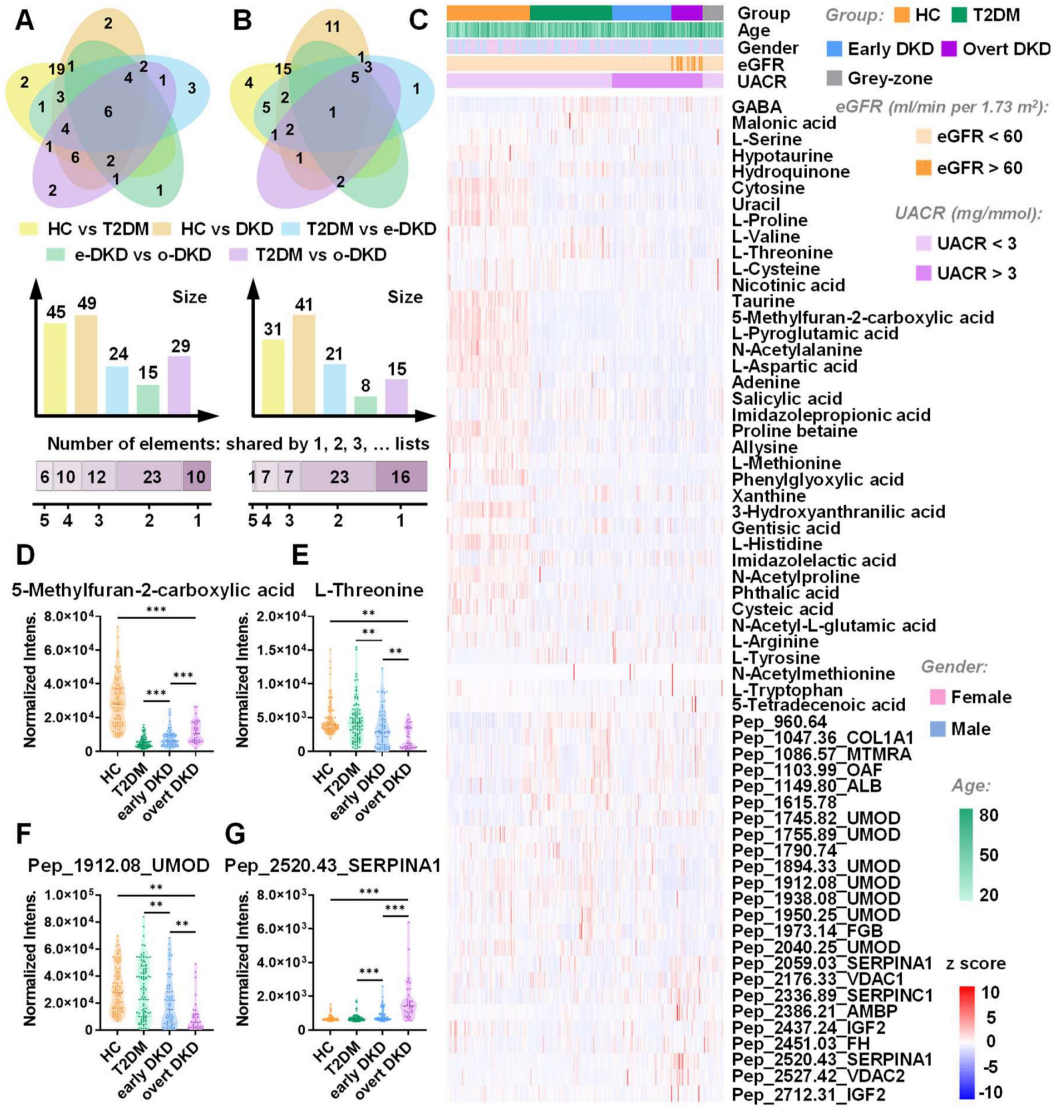
Multi-omics landscapes of urine samples from T2DM, DKD patients and healthy controls. (A) Venn diagrams represent differential metabolites and (B) peptides discovered in pairwise analysis, respectively. (C) Heatmap displays the distribution of differentially expressed features across three groups in the discovery set. Each row represents a signature whereas each column represents a sample. (D) Representative box plots of top up- or down-regulated metabolites and (G) peptides.

To obtain the comprehensive metabolomic landscape of urine samples, untargeted metabolic profiling was conducted. The detailed demographic information of collected urine cases for model establishment (152 HC,149 T2DM, 106 early DKD, 55 overt DKD, and 39 grey-zone patients) is shown in Table S1. The discovery and validation sets were retrospectively and randomly created in 2:1 proportion. Through untargeted LDI-MS analysis, around 227 metabolite peaks and 206 peptide peaks could be reliably detected across all samples. As shown in Figure S14, MS spectra obtained from HC, T2DM and DKD patients with different stages displayed distinct metabolome and peptidome landscapes. Venn diagram showed the overlap of differential metabolites and peptides in pairwise analysis between groups (Figure 1A-B, Table S2-S4). Notably, 10 metabolites and 6 peptides with adjusted p value < 0.05 were found to be significantly altered across all DKD groups, which might be intimately associated with DKD occurrence (Figure S15). The distribution of these differentially expressed features among the samples in the discovery set was presented in Figure 1C. In this study, we observed a phenomenon that most metabolites in the T2DM/DKD group were downregulated. Perturbations of metabolites involved in amino acid metabolic pathways might mediate the occurrence and development of diabetes associated with insulin resistance and dysfunction of pancreatic islet β-cells 28,29. Compared to HC, a majority of peptides displayed an up-regulation in patients, which may be explained by the reduction of glomerular filtration rate 30,31. Besides, Figure 1D-G displayed the top up- and down-regulated stepwise metabolites and peptides, including 5-Methylfuran-2-carboxylic acid, L-threonine, pep_2520.43_SERPINA1, and pep_1912.08_UMOD. The detailed information for the identification of biomarker candidates is described in Table S5-S7. The log2-transformed fold change of certain molecules and clinical factors with corresponding p value in pairwise analysis were visualized in Figure S16. As shown in the Figure, eGFR changes were related with DKD progression, and UACR levels were significantly different between early DKD and T2DM controls. However, the current clinical biochemical indexes cannot clearly separate the three groups of T2DM, early DKD and overt DKD. In contrast, OPLS-DA based on metabolomics or peptidomics data achieved a certain separation between the three groups. especially for T2DM and overt DKD patients (Figure S17A-B). Interestingly, there was less overlap between T2DM and early DKD patients in the pattern recognition when integrating metabolomics and peptidomics (Figure S17C). To assess the impact caused by demographic characteristics within cohorts, we examined the discrimination outcomes across groups with different gender, age and BMI using both discovery and validation datasets. The PCA plots illustrated no significant differences in mass spectra of males and females from different subgroups and the interference caused by age and BMI variation could be ignored (Figure S18, S19).

### Multi-omics reveals dysregulated biological pathways in different disease stages

**Figure 2:**
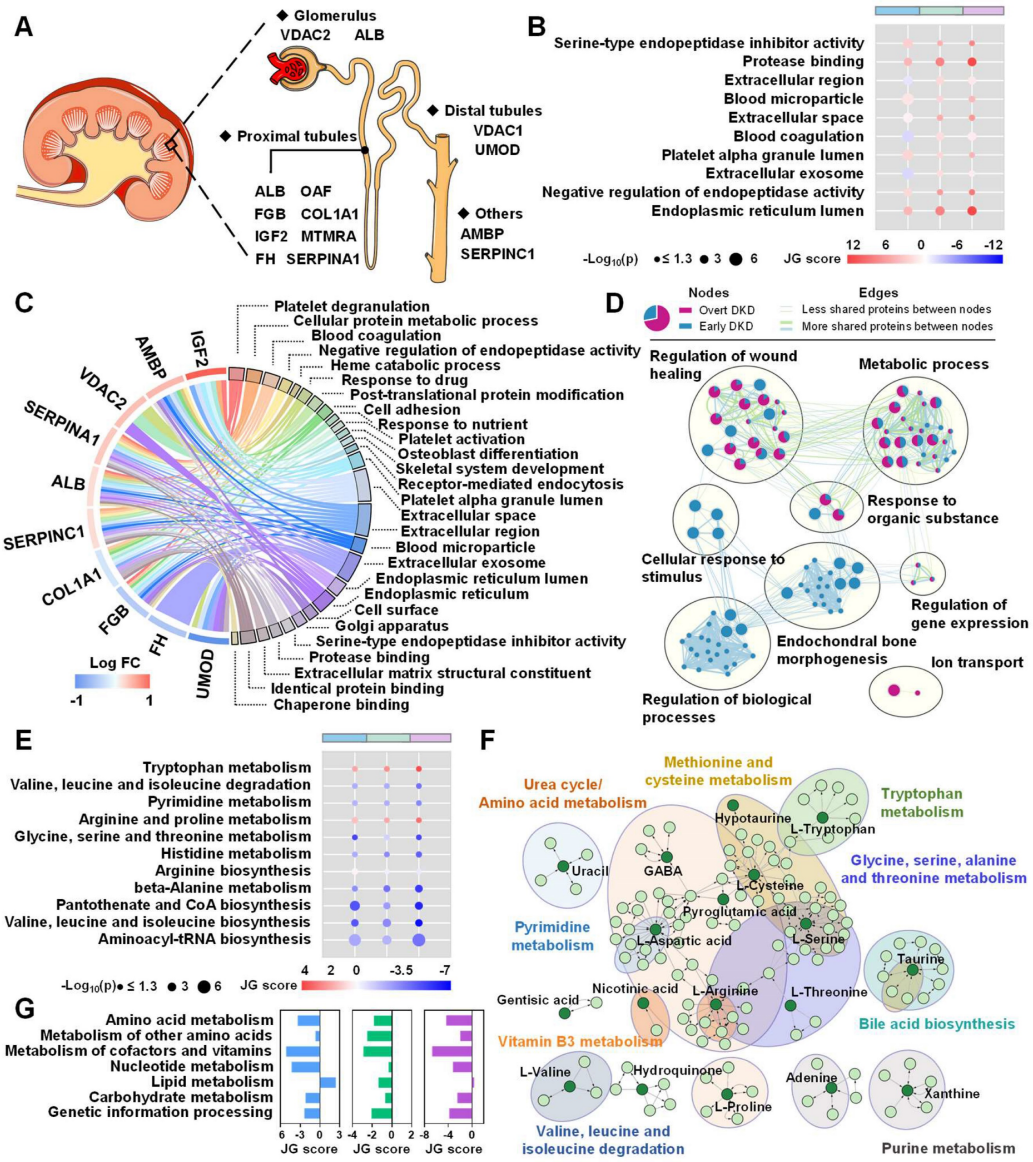
Dysregulated biological pathways in different disease stages. (A) Localization of assigned proteins in different nephron segments. (B) Common biological processes in different disease stages. The circle colors represent JG scores of certain dysregulated processes between two groups. The circle size is proportional to the log_10_(p) value. (C) Chord plot of top 10 ranked assigned proteins and associated biological pathways. (D) Comparison of enriched biological processes in different disease stages. As shown in the figure legend, biological processes enriched in early DKD compared to T2DM controls appear as blue clustered nodes, while purple node represents biological processes enriched in different DKD stages. (E) Common metabolic pathways in different disease stages. (F) Network analysis of identified metabolites shows the disturbance in amino acid metabolism. (G) Relative enrichment of the major metabolic pathways in pairwise analysis, represented by JG score. Blue, green and purple represent pairwise analysis between T2DM and early DKD, early DKD and overt DKD, T2DM and overt DKD, respectively.

The dysregulated biological pathways in different disease stages were further investigated by inputting those signatures into the open-source platforms as well as literature mining. As shown in Figure 2A, the altered urinary peptide-attributed proteins were mapped by the Human Protein Altas according to their expression in renal tissues. The differential excretion of tubular proteins such as UMOD suggests that the tubular compartment is an important site of early injury. Attributed proteins such as VDAC2 and ALB are localized in the glomerulus and proximal tubules, which may reflect impaired glomerular permselectivity and proximal tubular reabsorption. Biological function analysis indicated that the occurrence of early DKD may be related to cellular protein metabolic process, platelet alpha granule lumen, and serine-type endopeptidase inhibitor activity (Figure S20). Ten common biological processes were identified to be dysregulated across three groups by intersection pathway analysis (Figure S21) and the relative enrichment scores and significance of these processes between two groups were visualized in Figure 2B.

**Figure 3:**
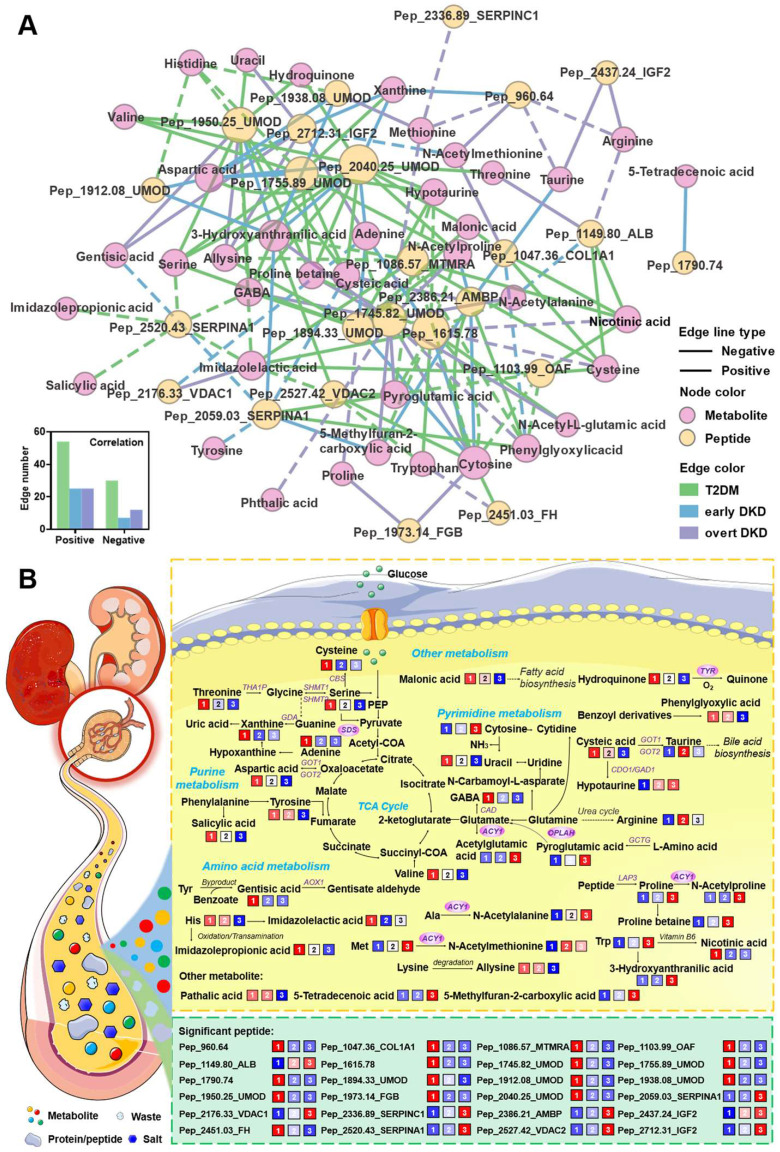
(A) Significant associations between differential metabolites and peptides in the T2DM, early DKD and overt DKD groups (absolute value of the correlation coefficient > 0.3, p < 0.05). The associations were estimated by biweight midcorrelations, and the corresponding Student p-values were calculated. The node colors represent the molecular types and the solid and dashed lines represent positive and negative correlations, respectively. The bottom-left bar plot summarizes the positive and negative edge numbers in different groups. (B) Pathway map of the differential metabolites and peptides. The levels of perturbed features in the three groups are visualized in color, and differentially expressed genes are marked with red circles.

The associations between these source proteins and the corresponding pathways are shown in Figure 2C. To compare the enriched biological processes during DKD onset and development, an enrichment network analysis was carried out. As shown in Figure 2D, the majority of processes involved in the DKD occurrence were also differ between two DKD stages, including regulation of wound healing, metabolic process, response to organic substance, and regulation of gene expression. To explore the impact of metabolite alterations in response to different stages of DKD, pathway and network analyses were performed on the basis of the differently expressed metabolites. Intersection pathway analysis confirmed that 11 common metabolic pathways were disturbed across three groups (Figure S22) and Figure 2E revealed that aminoacyl-tRNA biosynthesis, valine, leucine, and isoleucine biosynthesis, and pantothenate and CoA biosynthesis were ranked at the top. Besides, network analysis displayed the metabolite-metabolite associations among early DKD-related metabolites (Figure S23) and the significantly altered pathways in the network were presented in Figure 2F. To measure the perturbation of certain metabolic pathway category in different DKD stages, JG score was calculated and displayed in bar plot. As shown in Figure 2G, most metabolic processes are hindered as kidney damage progresses, which has been observed in numerous works 11. Finally, integrative pathway analysis was achieved by combining the metabolomics and peptidomics results. Heterogeneous molecular network was constructed to assess the variations of metabolite-peptide interactions, which may reflect the molecular compensatory mechanisms in different groups (Figure 3A). Here, we noticed that peptide_1938.08_UMOD showed a negative association with aspartic acid in the early DKD group, which disappeared in T2DM and overt DKD. Instead, peptide_1938.08_UMOD showed a negative and positive association with histidine and methionine in T2DM and overt DKD, suggesting the influence of disease-specific regulation mechanisms. Besides, the joint-pathway analysis revealed that differential metabolites and attributed proteins were involved in integrated pathways such as protein digestion and absorption (Figure S24A). A bar plot was pictured to display the representative perturbed processes (p < 0.05) enriched in metabolites, source proteins of the peptides or their combinations in different disease stages. As shown in Figure S24B, amino acid metabolism and complement as well as coagulation cascades were determined to be associated with the DKD phenotype. To validate the molecular mechanisms identified from urine samples, transcriptome analysis was performed based on RNASeq data from human kidneys with DKD and morphologically normal kidneys. As expected, 366 upregulated genes and 510 downregulated genes were differentially expressed in the DKD group, suggesting that there exists a dramatic molecular change during DKD pathogenesis (Figure S25). KEGG functional enrichment analysis revealed that the altered metabolic pathways were mainly focused on amino acid metabolism including protein digestion and absorption, biosynthesis of amino acids, tryptophan metabolism, arginine and proline metabolism, and so on (Figure S26A). Furthermore, Gene Ontology (GO) enrichment analysis indicated that dysregulated biological processes were concentrated in cellular protein metabolic process, platelet alpha granule lumen and so on (Figure S26B). Intriguingly, the disturbed metabolic pathways and biological processes observed in transcriptomics have been reflected in urine metabolomics and peptidomics in this work. What's more, mRNA expression analysis identified four metabolite-related genes were up-regulated in DKD patients, including *ACY1, OPLAH, SDS* and *TYR* (Figure S27). Finally, a pathway map was drawn to exhibit the perturbations of biomarker candidates and related genes during renal injury in diabetic patients (Figure 3B).

### Integrating peptidomics and metabolomics improves the diagnostic outcomes

**Figure 4:**
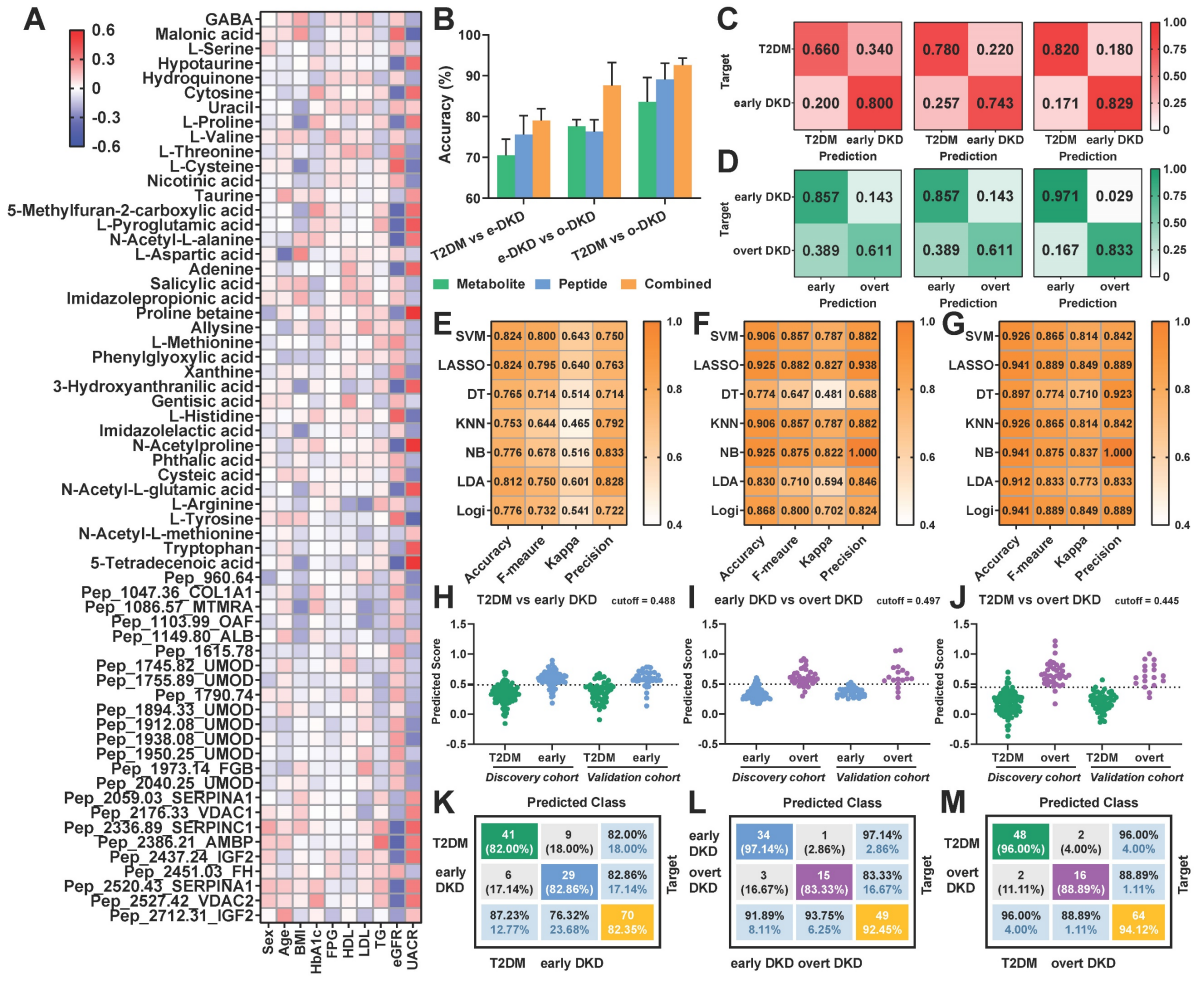
Pairwise predictions based on the differential signatures. (A) Heatmap visualizes the correlation of each feature with clinical baseline information. (B) The bar graph exhibits the average accuracy of the pairwise prediction models built on seven types of machine learning algorithms. (C) Heatmap of prediction accuracy for early DKD diagnosis and (D) DKD status discrimination based on metabolomics only (left), peptidomics (middle), and their combinations (right). (E) Performance of different machine learning algorithms for pairwise prediction in the validation dataset, evaluated by Kappa statistic, accuracy, F-measure, and precision for T2DM vs. early DKD, (F) early DKD vs. overt DKD and (G) T2DM vs. overt DKD. (H) Predicted scores of the LASSO regression model for early DKD diagnosis, (I) DKD status discrimination and (J) overt DKD diagnosis, respectively. (K) Confusion matrix for pairwise predictions based on LASSO regression model for T2DM vs. early DKD, (L) early DKD vs. overt DKD and (M) T2DM vs. overt DKD.

To determine whether combining peptidomics and metabolomics could enhance the accuracy of DKD diagnosis, pairwise predictions were performed. As shown in Figure 4A, the correlations between differentially expressed signatures and clinical parameters were systematically analyzed. Impressively, we found that 20 out of 62 potential biomarkers were associated with eGFR (| r | > 0.3) and UACR (| r | > 0.3) in patients. With the assistance of machine learning, prediction models were constructed on the basis of potential peptide markers alone, metabolite markers alone, and their combinations. Seven machine learning algorithms were investigated in this study, including SVM, DT, NB, Logi, LDA, KNN, and LASSO regression. Histograms were completed with the mean of accuracy from seven machine learning models and the results clearly indicated that the combined model significantly improved the average accuracy of early DKD diagnosis from 70.5% to 79.0% when compared to single-omics (Figure 4B, Figure S28A). Furthermore, enhanced diagnostic outcomes from the well-trained multi-omics models were also observed in overt DKD diagnosis with an average accuracy of 92.6% (Figure 4B). For the separation of early DKD and overt DKD group, nearly 76.3% of patients in the validation cohort were successfully predicted using peptidomics data alone, whereas the combined model increased the average prediction accuracy to 87.6% (Figure 4B).

Taking the LASSO regression model results as an example, heatmaps of the predicted ratios observed a significant reduction in the proportion of misclassification for both early DKD diagnosis and DKD status discrimination (Figure 4C-D, Figure S28B-C). These results suggested that multi-omics prediction holds greater potentials than peptidomics and metabolomics alone, especially for early DKD diagnosis. Noteworthy, four aspects were taken into consideration to unbiasedly evaluate the model performances of different classification methods, including accuracy, F-measure, kappa coefficient and precision. As shown in Figure 4E-G and Figure S28D-F, the LASSO regression model exhibited fairly good performance in early DKD diagnosis and DKD status prediction in both training and validation cohorts compared to other algorithms. Meanwhile, a prediction score can be calculated for disease assessment using the established LASSO regression formula. Therefore, we finally employed the LASSO regression algorithm to reduce data dimensions and dig out predictive biomarker candidates. The binary prediction outcomes of LASSO regression model for the discovery and validation cohorts were displayed in Figure 4H-M.

### Stepwise diagnostic model for diabetic kidney disease

**Figure 5:**
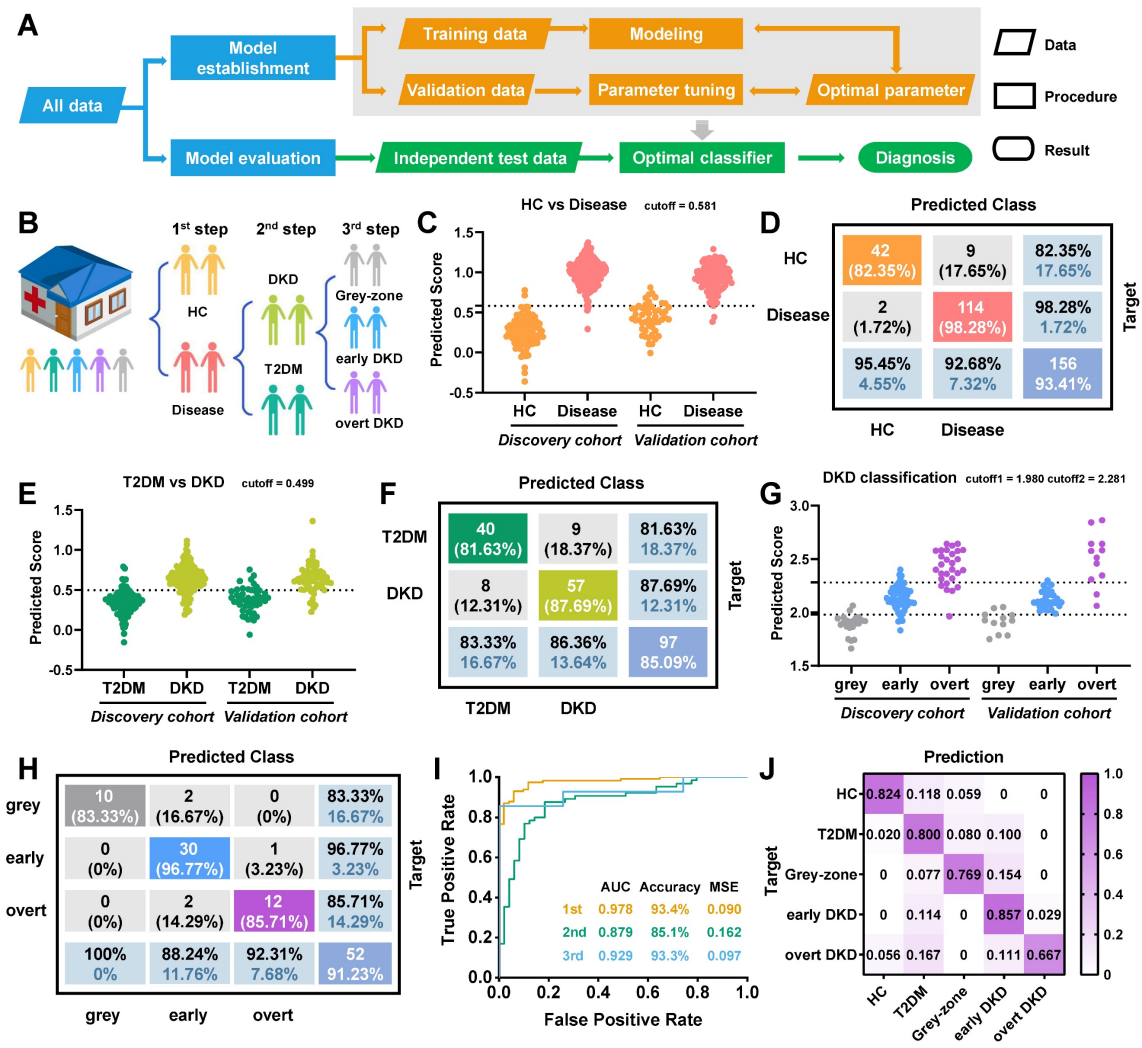
Three-step predictive model for DKD diagnosis. (A) Schematic overview of machine learning approach used to develop and validate the stepwise diagnostic model. (B) Concept of three-step predictive model. (C) Predicted scores for the first-layer diagnosis. (D) Confusion matrix for disease diagnosis in the validation set. (E) Predicted scores and (F) confusion matrix for the second-layer DKD diagnosis in the validation set, respectively. (G) Predicted scores for DKD status discrimination. (H) Confusion matrix for the third-layer prediction and (I) Receiver operating curves for the stepwise prediction in the validation set, respectively. (J) Heatmap displays the ratios of predicted cases to true cases in the verification cohort.

**Figure 6:**
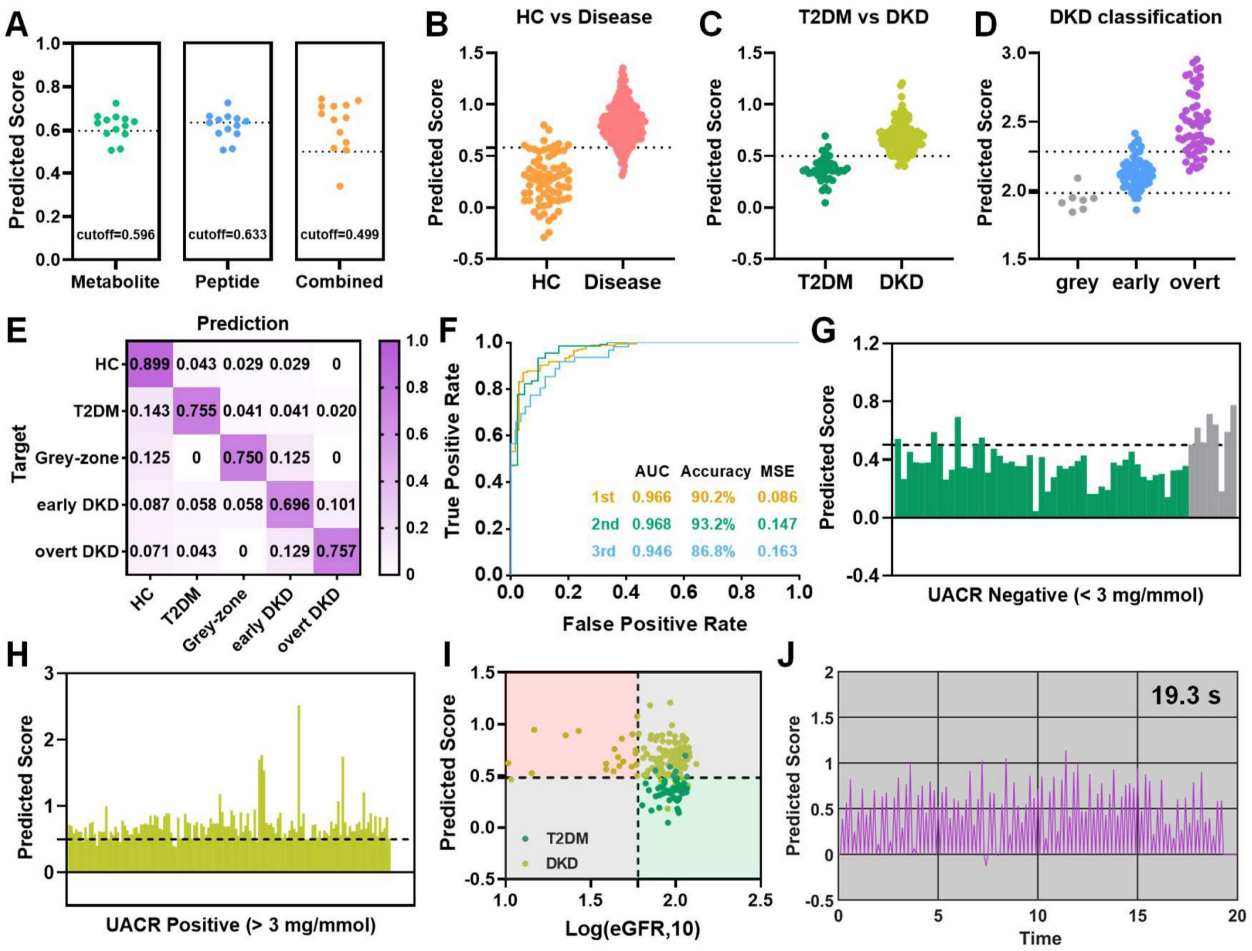
Diagnostic performance of the stepwise prediction model. (A) Prediction results of grey-zone samples based on single-omics and their combinations. (B) Predicted scores for the first, (C) second, and (D) third-layer classification, respectively. (E) The ratios of predicted cases to true cases for different groups and (F) receiver operating curves for the stepwise prediction in the external validation cohort, respectively. (G) Predicted scores for UACR-negative and (H) positive patients by multi-omics model, respectively. (I) 2D coordinate plot visualizes diagnostic results for each sample based on eGFR levels and established model. (J) The simulated process for the second-layer real-time DKD diagnosis. The simulation batch used here consists of 96 cases.

To address the challenges of early DKD diagnosis in high-risk diabetic populations and achieve DKD status prediction, a three-step diagnostic model was established using the altered metabolites and peptides. Schematic overviews of the machine learning approach and stepwise diagnostic model were illustrated in Figure 5A-B. Particularly, various classification and regression models have been systematically evaluated and the LASSO regression model was finally selected. For the first layer of predictive model, a biomarker panel consisting of 22 predictive marker candidates was established to distinguish healthy controls from patients with T2DM or diabetic kidney disease, including malonic acid, pep_1047.36_COL1A1 and so on. As shown in Figure 5C-D and Figure S29A, the model achieved an excellent diagnostic performance for disease group, with a sensitivity of 98.28% and specificity of 82.35% in validation sets. Subsequently, the samples diagnosed as diseases were input into the second-layer model to conduct an accurate diagnosis, distinguishing DKD (grey-zone, early DKD and overt DKD) from T2DM controls. A biomarker panel consisting of 23 differential features related to the DKD phenotype was established and the prediction scores for the discovery cohort of 229 cases and the validation cohort of 114 cases were provided in Figure 5E. Only 10.42% of T2DM subjects in the discovery set were misclassified, while 87.69% of DKD samples in the verification set were successfully picked out (Figure 5F, Figure S29B). Finally, DKD patients were input into the third-layer LASSO regression model for status classification. The combination of 3-hydroxyanthranilic acid, histidine, malonic acid, proline, proline betaine, uracil, pep_1103.99_OAF, pep_1912.08_UMOD, pep_2176.33_VDAC1, pep_2386.21_AMBP, and pep_2520.43_SERPINA1 was defined as the ideal biomarker panel to distinguish early DKD patients from grey-zone and overt DKD patients. The prediction scores and confusion matrix for DKD status classification in the training and validation cohorts were provided in Figure 5G, H and Figure S29C-D. In the verification dataset, the AUC values for the first-, second- and third-layer predictions were 0.978, 0.879, and 0.929, respectively (Figure 5I). In addition, the ratios of the predicted outcomes to actual cases were visualized in Figure 5J and Figure S29E. The results indicated that the subjects in the verification batch were classified with accuracy of 82.4% for HC, 80.0% for T2DM, 76.9% for grey-zone, 85.7% for early DKD and 66.7% for overt DKD (Figure 5J). Surprisingly, we found the multi-omics model could effectively identify grey-zone patients with an abnormal urinary microalbumin concentration in the UACR-negative population. 92.3% of grey-zone patients were distinguished from T2DM patients, indicating the possibility of applying the multi-omics technology in diabetes health management (Figure 6A). If using the well-trained single metabolomics model with a cutoff value of 0.596, the prediction accuracy for grey-zone patients was only 53.8%. As to the peptidomics model with a cutoff value of 0.633, the prediction accuracy for grey-zone samples was 61.5%. the diagnostic outcomes were further compared with recognized clinical indexes. To verify the reliability of established model, another independent external cohort was tested, which included 69 HC, 49 T2DM, 69 early DKD, 70 overt DKD and 8 grey-zone subjects (Table S1). As shown in Figure 6B-D and Figure S30, 90.31% of individuals diagnosed with disease were correctly picked out and 94.81% DKD patients were successfully identified by the stepwise diagnostic model. Overall, the subjects in the independent external cohort were classified with correction rates of 89.9% for HC, 75.5% for T2DM, 75.0% for grey-zone, 69.6% for early DKD and 75.7% for overt DKD (Figure 6E). As shown in Figure 6F**,** AUC values from the ROC curves that differentiate disease from HC, T2DM from DKD, and early DKD from others were 0.966, 0.968, and 0.946, showing excellent diagnostic performance. Nearly 87.5% of grey-zone patients in the external validation cohort were distinguished from controls and 94.96% UACR-positive patients had enough high predicted scores to separate from T2DM group (Figure 6G, H). For the DKD group of external validation dataset, only 15.65% of patients had an eGFR value less than 60 ml/min per 1.73 m^2^. As shown in Figure 6I, compared with eGFR index, the prediction results based on multi-omics model display significant improved diagnostic accuracy for DKD patients.

With the aid of computer technology, we envisioned that this well-trained LASSO regression model could return automatic, real-time prediction results of DKD diagnosis. Therefore, a simulation for the complete workflow from data acquisition to the final prediction was performed using a batch sample containing 96 cases. Figure S31A showed the file conversion, peak read and data normalization can be completed within a few seconds. Afterward, the nearly real-time prediction was carried out by the pre-deployed stepwise LASSO regression model in Simulink (Figure S31B). Figure 6J and Figure S31C displayed the simulated real-time diagnosis processes for each step predictions, which only takes average 0.2s per sample. Collectively, the multi-omics model may serve as a promising and robust approach for early DKD screening and DKD status classification.

## Discussion

Diabetic kidney disease has attracted widespread attention as the leading cause of kidney failure worldwide. Unfortunately, the “gold standard” histopathological examination has not been established due to the reluctance of practitioners to biopsy patients with diabetes. Currently, UACR is widely accepted in clinical practice to reflect the DKD progression. Nevertheless, no significant increase in UACR levels was observed in 20% of T2DM patients with reduced glomerular filtration rate 8. In addition, common comorbidities of T2DM such as hypertension or obesity may also impair the glomerular filtration barrier, resulting in insufficient sensitivity and specificity of microalbuminuria in DKD diagnosis 32. Estimated GFR is recognized as the predictor of chronic kidney disease, but its application is limited by the difficulty of obtaining a patient's baseline GFR and the fact that GFR changes often appear in the late stages of renal insufficiency 33. Therefore, mining new biomarkers for DKD diagnosis is of great significance for early treatment and improved prognosis.

Driven by the development of high-throughput technologies, peptidomics and metabolomics have been rapidly applied to specific clinical settings. An increasing body of evidence has suggested that a panel composed of multiple biomarkers exhibits great priorities in improving accuracy, enhancing diagnosis, and reducing misclassifications 18,19. Analysis of native or endogenous peptides in biological fluids can provide valuable insights into disease mechanisms. In addition, significant peptides can be served as potential biomarkers for the non-invasive monitoring of various diseases such as renal injury. It is well known that diabetic kidney disease is a complex metabolic disease with no specific clinical symptoms in the early stage. Metabolomic analysis in clinical studies and animal models have revealed that metabolic pathways such as amino acid metabolism and tricarboxylic acid cycle have been altered in the early DKD 11,34,35. Therefore, integrative omics is expected to provide an unprecedented view of understanding DKD mechanisms and discovering potential biomarkers that enable effective early DKD diagnosis.

In this work, six peptides and ten metabolites were verified to be stepwise regulated in different DKD stages. Here, we elucidated the role of the corresponding proteins and metabolites in the DKD mechanisms through literature surveys and database searches. AMBP belongs to the lipoprotein transporter superfamily, the increased urinary AMBP levels were assumed to correspond to proximal tubular dysfunction, which has been confirmed in numerous earlier studies 30,36. IGF2 plays important physiological roles in cell proliferation, energy maintenance and metabolic homeostasis. It has been reported that the expression of IGF2 and IGF2BP2 genes is markedly altered in DKD 37. SERPINC1 and SERPINA1 are regulators of serine protease activity, and their marked upregulation may be associated with dysregulation of many biological processes during DKD pathogenesis, such as hemagglutination, complement activation, and inflammation 38. UMOD is exclusively synthesized in the kidney, which participates in ion transport, electrolyte balance, kidney innate immunity and so on 39. Consistently, our study also indicated that lower UMOD fragments levels are strongly connected with kidney damage in diabetic patients. VDACs constitute the major gateway for substance transport between the cytoplasm and mitochondria. Studies have indicated that the increased levels of VDACs proteins under hyperglycemic conditions would result in cell death due to energy starvation, which ultimately leads to organ complications such as cardiovascular disease, kidney disease, and stroke 40. The intersection of pairwise analysis also revealed differential metabolites associated with DKD pathogenesis and development, including malonic acid, serine, hydroquinone, cytosine, uracil, proline, valine, threonine, 5-methylfuran-2-carboxylic acid and imidazolepropionic acid. These metabolic perturbations may be explained by absolute nephron loss, which is reflected in increased blood urea and creatinine levels. The decreased levels of malonic acid were assumed to correspond to the disorder of fatty acid biosynthesis. Several compounds related to pyrimidine biosynthesis were significantly dysregulated in the DKD group, including cytosine and uracil 41. Furthermore, we observed that most amino acids are decreased during renal injury, which has been confirmed in numerous works 41,42. What's more, the intersection of enriched pathways revealed that 14 common metabolic pathways are disturbed in different DKD stages, mainly focusing on amino acid metabolism. Surprisingly, renal transcriptomics confirmed perturbations in amino acid biosynthesis and amino acid metabolism found in urine metabolomics, such as arginine biosynthesis and glycine, serine and threonine metabolism. Joint-pathway analysis suggested that differential metabolites and attributed proteins are jointly involved in the DKD-related biological changes such as protein digestion and absorption, which provides a new direction for us to further understand the DKD mechanism.

Impressively, integrative omics resulted in improved outcomes in early DKD diagnosis and DKD status discrimination compared to single omics. To verify our original hypothesis that the combination of potential peptide and metabolite biomarkers may hold potential in monitoring the onset of early-stage DKD, we screened different machine learning algorithms and the LASSO regression algorithm was finally taken into consideration. Interestingly, the multi-omics model for early DKD diagnosis reduced the false prediction rate by nearly 10% compared to the single omics in the validation cohort. At the same time, the combined model built with LASSO regression algorithm showed considerable advantages in DKD status classification, increasing the general accuracy from 76.3% to 87.6%. Therefore, a stepwise prediction model for DKD diagnosis was constructed based on the integration of urine peptidomics and metabolomics. With the aid of the well-trained prediction model, 89.9%, 75.5%, 69.6%, and 75.7% of HC, T2DM, early DKD and overt DKD patients in the external verification set were successfully identified, respectively. Compared with eGFR (< 60 ml/min per 1.73 m^2^), the constructed model improved the diagnostic accuracy of DKD patients from 15.65% to 94.81%. Amazingly, the established model can pick out 75% of the grey-zone samples with abnormal urinary microalbumin concentration. Compared with previous serum multi-omics studies, this stepwise model significantly improved the diagnostic accuracy of early DKD from 42.6% to 69.6%, demonstrating the potential of urine as an "end-product recycler" in the DKD diagnosis 19. Although the diagnostic accuracy of our model is not much different from that of the reported CKD273 classifier, we believe that this multi-omics model based on the LDI-MS platform can obtain DKD-related molecular information in a few seconds, which is expected to provide a new way for rapid screening of large populations in the future 43. What's more, the possibility of our model returning nearly real-time predictions in large-scale populations has been explored by the simulation built in Simulink.

## Conclusion

In general, the novel aspects of this study mainly lie in the construction of a high-throughput multi-omics platform and the integration of urine peptidomics and metabolomics to enhance the DKD diagnosis, especially for early-stage DKD. Fairly good reproducibility was obtained for urine metabolic fingerprints and peptide profiles, which provided a basis for subsequent biomarker discovery and disease identification. Integrative omics model constructed with machine learning algorithm significantly increased the diagnostic outcomes for T2DM and all stages of DKD. Totally 85.5% of early DKD patients were correctly separated from T2DM volunteers and healthy controls in the external validation set, confirming the feasibility as a diagnostic method for early DKD. Besides, 85.71%, 81.36%, and 85.48% of grey-zone, early DKD and overt DKD patients were successfully identified in the third-layer model, so we speculate that the constructed model can assist the current clinical metrics to achieve a more accurate DKD status discrimination. Last but not least, 87.5% of grey-zone patients missed by UACR and eGFR metrics were successfully picked out from T2DM patients, illustrating the promise of our strategy to enable earlier DKD surveillance. Perturbed metabolic pathways such as amino acid metabolism and biological processes such as the cellular protein metabolic process were validated by renal transcriptomics. The four metabolite-related candidate genes identified in this work are expected to become potential drug targets, including *ACY1, OPLAH, SDS* and* TYR*. Based on the above results, we are confident that this integrative approach may provide a new choice for personalized DKD care in the future. Despite these promising results, the current study has several limitations: 1) Renal damage may have occurred in T2DM patients with normal microalbuminuria, and subsequent follow-up in T2DM group will help us to discover valuable signatures for early DKD prediction. 2) Urine samples from non-diabetic chronic kidney disease were not included in this study. Therefore, whether metabolite or peptide perturbations in DKD patients are owing to renal damage or diabetes itself should be evaluated in future studies. In the next five years, we will enroll subjects from multiple centers and conduct follow-up in the T2DM group, hoping to identify valuable features for early prediction of DKD.

## Supplementary Material

Supplementary experimental procedures, supplementary results and discussion, and MS/MS identification of metabolites and peptides.

## Abbreviations

T2DM: type 2 diabetes mellitus
DKD: diabetic kidney disease
LDI-MS: laser desorption/ionization mass spectrometry
MALDI-TOF MS: matrix-assisted laser desorption/ionization time-of-flight mass spectrometry
PCA: principal component analysis
OPLS-DA: orthogonal partial least squares-discriminant analysis
eGFR: estimated glomerular filtration rate
UACR: urinary albumin-to-creatinine ratio
mALB: microalbumin
KEGG: Kyoto Encyclopedia of Genes and Genomes
