# Supplementary material for: Integration of metabolomics and peptidomics reveals distinct molecular landscape of human diabetic kidney disease
Source: Theranostics. 2023 May 21;13(10):3188–203. doi: 10.7150/thno.80435 (PMC10283058; doi:10.7150/thno.80435)
Supplement: Supplementary file 1 — Supplementary experimental procedures, supplementary results and discussion, and MS/MS identification of metabolites and peptides. [file thnov13p3188s1.pdf]

Electronic Supplementary Material (ESI) for Theranostics.

Electronic Supporting Information

Integration of metabolomics and peptidomics reveals distinct molecular landscape of human diabetic kidney disease

Xinrong Jiang[a], Xingyue Liu[a], Xuetong Qu[a], Pingya Zhu[d], Fangjie Wo[d], Xinran Xu[a], Juan Jin[,c], Qiang He[*b,c] and Jianmin Wu[*,a]*

Table of Supporting Information Contents:

1. Supplementary Experimental Procedures

1.1 Sample size calculation

1.2 The detailed experimental parameters of UPLC-MS analysis

1.3 The detailed experimental parameters of nano-LC-ESI MS/MS analysis

1.4 The detailed procedure of transcriptome analysis

2. Supplementary Figures

Fig.S1 The orthogonal partial least squares discrimination analysis (OPLS-DA) for QC sample and clinical samples involved in this project.

Fig.S2 Reproducibility of the multi-omics platform.

Fig.S3 Monitoring the fluctuations of the internal standard ratios in urine samples.

Fig.S4 Standard addition curves of metabolites in artificial urine samples detected by tip-contact extraction method.

Fig.S5 Standard addition curves of peptides in artificial urine samples detected by pSi-based technology.

Fig.S6 Recovery yield of nine metabolites in artificial urine samples using TCE technique.

Fig.S7 Recovery yield of four peptides in artificial urine samples using pSi-based technology.

Fig.S8 Accuracy of nine metabolites in artificial urine samples using TCE technique.

Fig.S9 Accuracy of four peptides in artificial urine samples using pSi-based technology.

Fig.S10 Precision of nine metabolites in artificial urine samples using TCE technique.

Fig.S11 Precision of four peptides in artificial urine samples using pSi-based technology.

Fig.S12 Dilution effect and stability of urine metabolomics.

Fig.S13 Dilution effect and stability of urine peptidomics.

Fig.S14 Representative LDI mass spectra of urine samples collected from HC, T2DM and DKD patients.

Fig.S15 Box plots of the normalized intensities of stepwise differential metabolites and peptides in the HC, T2DM, early DKD and overt DKD groups.

Fig.S16 The circle colors represent the log2-transformed fold change of certain molecules or

clinical factors between two groups.

Fig.S17 The OPLS-DA result for discrimination between T2DM, early DKD and overt DKD.

Fig.S18 The PCA score plot for metabolic discrimination of individuals with different age, BMI, and gender between different subgroups.

Fig.S19 The PCA score plot for peptide distinction of individuals with different age, BMI, and gender between different subgroups.

Fig.S20 Functional enrichment analysis based on dysregulated peptides, including biological process (BP), cellular components (CC) and molecular function (MF).

Fig.S21 Intersection analysis of enriched biological processes related to DKD onset and progression.

Fig.S22 Intersection analysis of enriched pathways related to DKD onset and progression.

Fig.S23 Metabolite-metabolite interaction network based on early DKD-related metabolites.

Fig.S24 Joint-pathway analysis.

Fig.S25 Volcano plot showing the differential expressed genes human kidneys with DKD and morphologically normal kidneys.

Fig.S26 Gene ontology and KEGG pathway enrichment analysis of differential genes screened out by edgeR package.

Fig.S27 The expression of candidate genes in human kidneys with DKD and morphologically normal kidneys from open database.

Fig.S28 Pairwise analysis based on different machine learning methods in the discovery cohort.

Fig.S29 Diagnostic performance of the stepwise prediction model in the discovery cohort.

Fig.S30 Confusion matrix for the stepwise diagnosis in the external validation set.

Fig.S31 Pipeline of nearly real-time molecular diagnosis by multi-omics platform using the established model.

3. Supplementary Tables

Table S1. Clinical characteristics of the subjects in this study.

Table S2. Summary of feature peaks that could be used to distinguish early DKD patients from T2DM subjects.

Table S3. Summary of feature peaks that could be used to distinguish early DKD patients from

overt DKD subjects.

Table S4. Summary of feature peaks that could be used to distinguish overt DKD patients from T2DM subjects.

Table S5. LC-MS/MS identification of potential metabolite peaks for discrimination of patients with diabetic kidney disease.

Table S6. MALDI-TOF/TOF tandem mass spectrometry identification of urine metabolites with commercial standard reagents.

Table S7. Nano-LC-ESI MS/MS identification of urine peptides.

1. Supplementary Experimental Procedures

1.1 Sample size calculation

A reasonable sample size of external validation cohort was calculated according to the formula:

$$n = \left(\frac{Z_{1-\alpha/2} \times \sqrt{p \times (1-p)}}{\delta}\right)^2$$

Where n represents sample size, p represents the expected sensitivity or specificity, Z represents tests statistic, δ represents the allowable error of sensitivity or specificity.

For HC vs Disease (T2DM + DKD), the parameters are set as follows:

Specificity: 85% ± 10%; Sensitivity: 90%±10%; δ: 0.1

Significant level (α) 0.05; Confidence (1-α): 0.95; Z1-α/2: 1.96

test type: two-sided test

$$n_{sp} = \left(\frac{1.96 \times \sqrt{0.85 \times (1-0.85)}}{0.1}\right)^2 = 49 \quad n_{se} = \left(\frac{1.96 \times \sqrt{0.9 \times (1-0.9)}}{0.1}\right)^2 = 35$$

That is, 49 cases of HC and 35 cases of disease were required.

For T2DM vs early DKD, the parameters are set as follows:

Specificity: 85% ± 10%; Sensitivity: 85% ± 10%; δ: 0.1

Significant level (α) 0.05; Confidence (1-α): 0.95; Z1-α/2: 1.96

test type: two-sided test

$$n_{sp} = \left(\frac{1.96 \times \sqrt{0.85 \times (1-0.85)}}{0.1}\right)^2 = 49 \quad n_{se} = \left(\frac{1.96 \times \sqrt{0.85 \times (1-0.85)}}{0.1}\right)^2 = 49$$

That is, 49 cases of T2DM and 49 cases of early DKD were required.

For early vs overt DKD, set the parameters to: Overt DKD

Specificity: 85%±10%; Sensitivity: 90%±10%; δ: 0.1

Significant level (α) 0.05; Confidence (1-α) : 0.95; Z1-α/2: 1.96

test type: two-sided test

$$n_{sp} = \left(\frac{1.96 \times \sqrt{0.85 \times (1-0.85)}}{0.1}\right)^2 = 49 \quad n_{se} = \left(\frac{1.96 \times \sqrt{0.9 \times (1-0.9)}}{0.1}\right)^2 = 35$$

That is, 49 cases of early DKD and 35 cases of overt DKD were required.

Overall, the minimum required sample size and actual sample size of the external validation cohort were listed as below:

Size	HC	T2DM	Early DKD	Overt DKD
Minimum	49	49	35	35
Actual	69	49	69	70

Note: Due to the rarity of clinical grey-zone patients, we only recruited 8 grey-zone subjects for external validation in the past few months.

1.2 The detailed experimental parameters of UPLC-MS analysis

Here, the detailed procedure of UPLC-MS analysis of urine samples were described. Before analysis, urine sample was prepared by pooling equal aliquots from all samples included in this study. Subsequently, the urine sample (200 μL) was mixed with acetonitrile (200 μL) to precipitate proteins. The mixture was vortexed and centrifuged at 14000 g for 10 min (4 ℃) to remove insoluble residues. After vacuum drying, the extracts were resuspended with 2% acetonitrile (200 μL). Of note, a 10K molecular weight ultrafiltration centrifuge tube was used to remove macromolecules.

The sample analysis was conducted on ACQUITY UPLC® HSS C18 column (2.1×100 mm, 1.8 μm, Waters, Milford, USA) in both positive and negative ionization modes. The injection volume was 5 μL. For RPLC separation, mobile phases A and B were 0.1% formic acid-water and acetonitrile, respectively. The flow rate of the mobile phase was 0.5 mL/min and the temperature was maintained at 40 ℃. Mass spectrometry experiments were performed on an orthogonal accelerated time of flight mass spectrometer (Waters, Milford, USA) equipped with an electrospray ion source. Scans from 50 to 1000 m/z at a resolution of 60,000 were used to acquire the full mass spectra.

1.3 The detailed experimental parameters of nano-LC-ESI MS/MS analysis

Before being transferred to the autosamplers, pooled urine sample was filtered through a 10K ultrafiltration centrifuge tube to remove macromolecules above 10 kDa. The filtrate was collected and analyzed on a Q Exactive HF-Orbitrap mass spectrometer (Thermo Scientific™, San Jose, USA) that was coupled to the nanoflow DIONEX UltiMate 3000 RSLCnano LC system in data dependent acquisition (DDA) mode. Buffer A was 2% ACN, 98% H_2O containing 0.1% formic acid (FA), and buffer B was 98% ACN in water containing 0.1% FA. All reagents were MS grade. For each acquisition, peptides were loaded onto a precolumn (3 μm, 100 Å, 20 mm×75 μm) at a flow rate of 6 μL/min for 4 min and then injected using a 60 min LC gradient (from 7% to 30% buffer B) at a flow rate of 300 nL/min (analytical column, 1.9 μm, 120 Å, 150 mm×75 μm). The MS full scan was acquired at a resolution of 60,000 with a scan range of 400 -1200 m/z. The automatic gain control (AGC) target was set to 3e6, and the maximum ion injection time (max IT) was set as 80 ms. The MS/MS scans were acquired at a resolution of 30,000. The AGC target is 1e5 and max IT was set as 100 ms. The first mass was set to 120 m/z and the isolation window was set to 1.6 m/z.

1.4 The detailed procedure of transcriptome analysis

The transcriptomics profiles of glomeruli from human kidneys with DKD and morphologically normal kidneys were obtained from the online GEO database (https://www.ncbi.nlm.nih.gov/geo/). Statistical analysis was performed using R 3.5.2 software and the differentially expressed genes were screened out by the R edgeR package. Statistical significance was defined as $p < 0.01$ and $|\log_2 FC| > 1$. Gene ontology and KEGG pathway enrichment analysis were performed by online KOBAS database (http://kobas.cbi.pku.edu.cn/kobas3).

2. Supplementary Figures

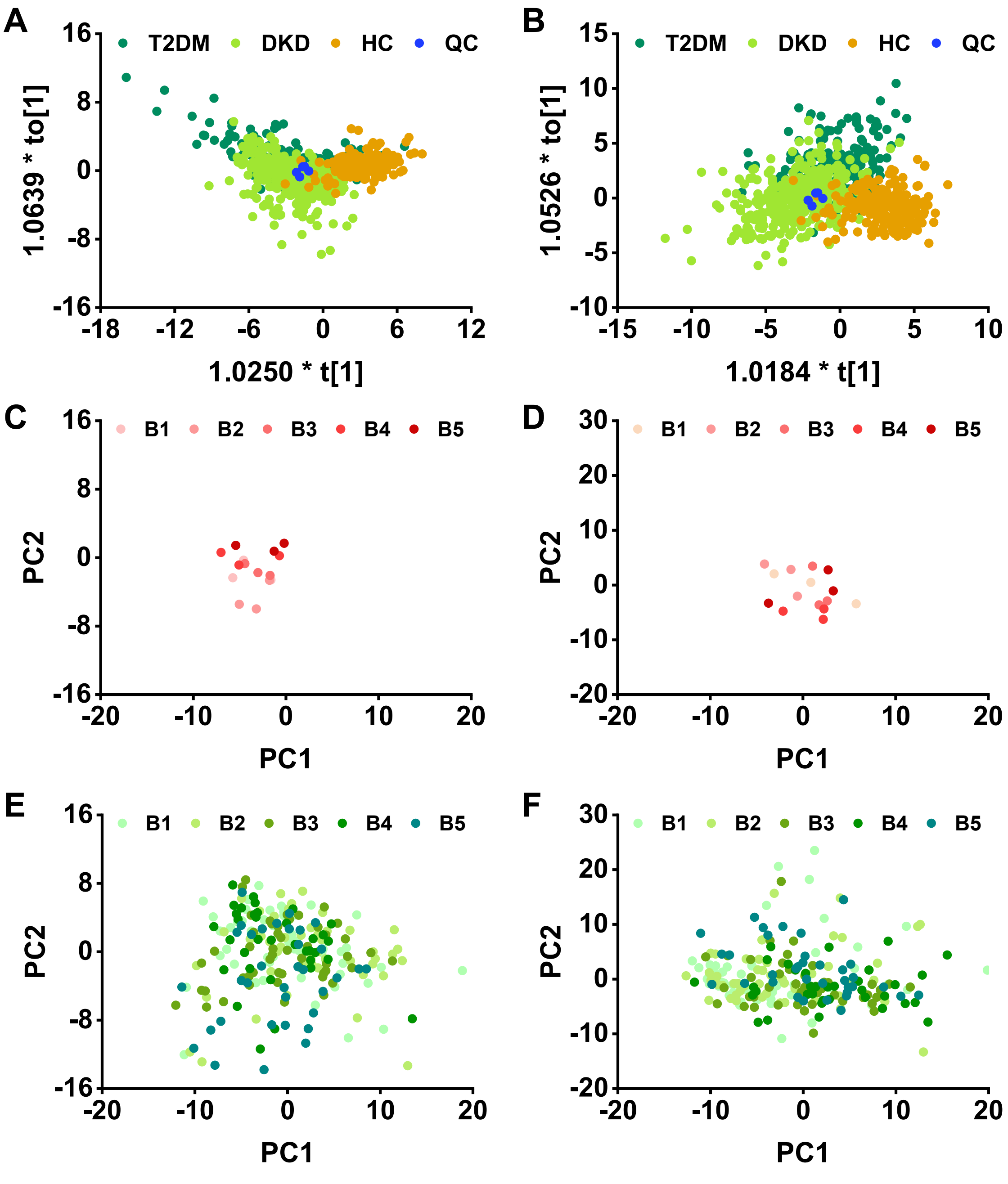

Figure S1 (A, B) The orthogonal partial least squares discrimination analysis (OPLS-DA) plot for QC sample and clinical samples involved in this project. (C, D) The principal components analysis (PCA) plot for QC sample during the five-batch test. (E, F) The PCA plot for healthy volunteers during the five-batch test. B1 to B5 represent five different batches. (A, C, E) Score plot based on metabolic fingerprints. (B, D, F) Score plot based on peptide profiles.

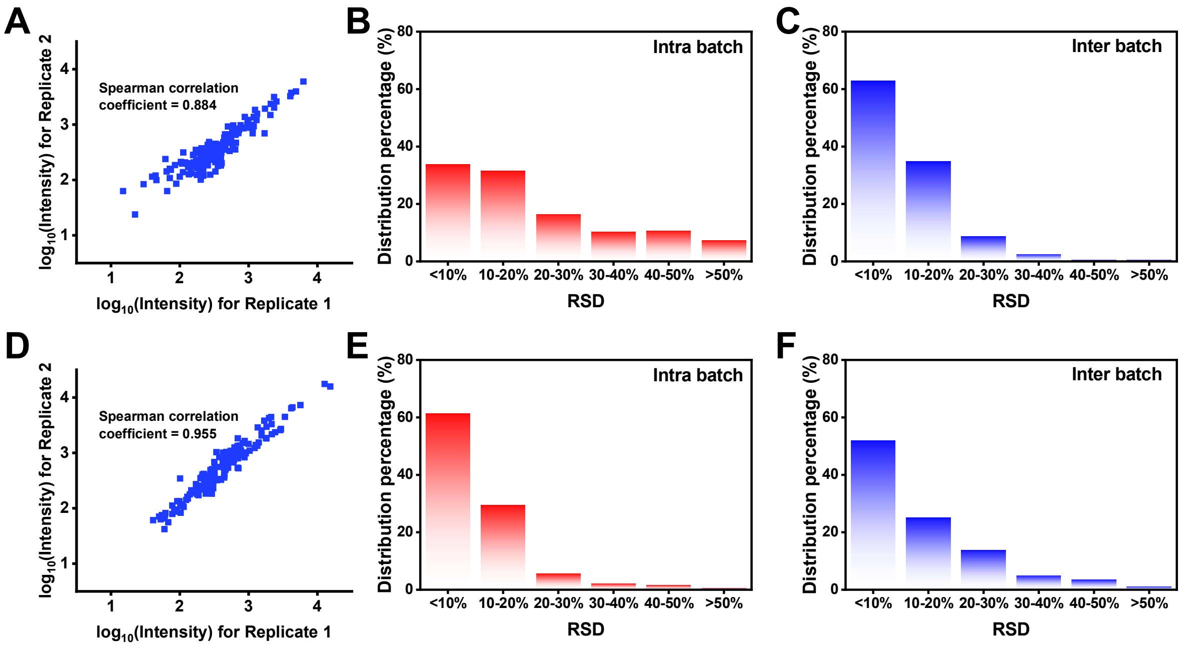

Figure S2 Reproducibility of the multi-omics platform. Metabolite and peptide abundances detected in pooled QC urine samples over two technical replicates are shown in (A) and (D), respectively. (B)(E) Intra-batch and (C)(F) inter-batch stability for metabolite and peptide profiles in QC sample, respectively.

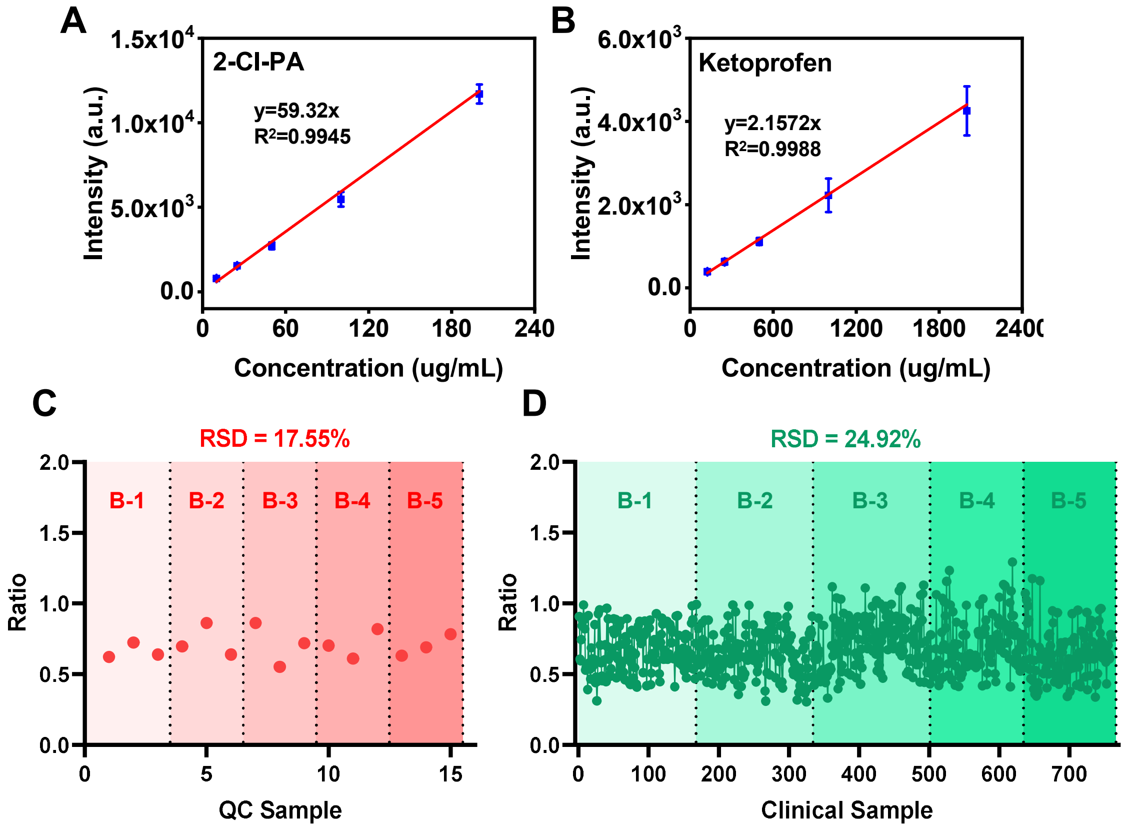

Figure S3 Monitoring the fluctuations of the internal standard ratios in urine samples. (A)(B)

Linearity of 2-Chlorophenylalanine and ketoprofen spiked in QC urine samples using tip-contact extraction technique, respectively. (C) Variation of internal standard peak ratios in the QC samples. Three replicates were performed in each batch of QC sample, and five batches of QC samples were tested during the whole investigation procedure. (D) Clinical urine samples during the five-batch test. B-1 to B-5 represent five different batches. The acquired MS data were processed by the cubic spline normalization method.

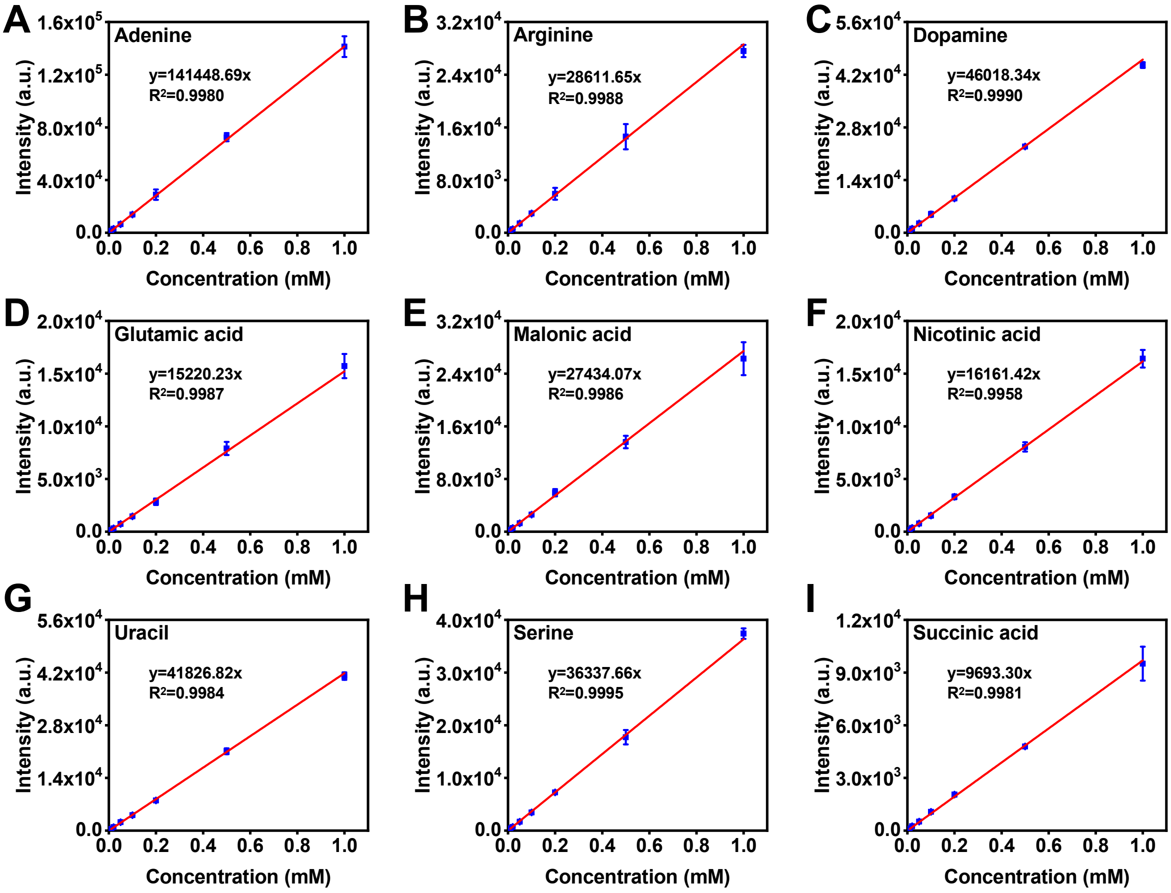

Figure S4 Standard addition curves of (A) Adenine, (B) Arginine, (C) Dopamine, (D) Glutamic acid, (E) Malonic acid, (F) Nicotinic acid, (G) Uracil, (H) Serine and (I) Succinic acid in artificial urine samples detected by tip-contact extraction method. Good linearities were obtained for standard-added artificial urine samples, ranging between 0.01 mM and 1 mM.

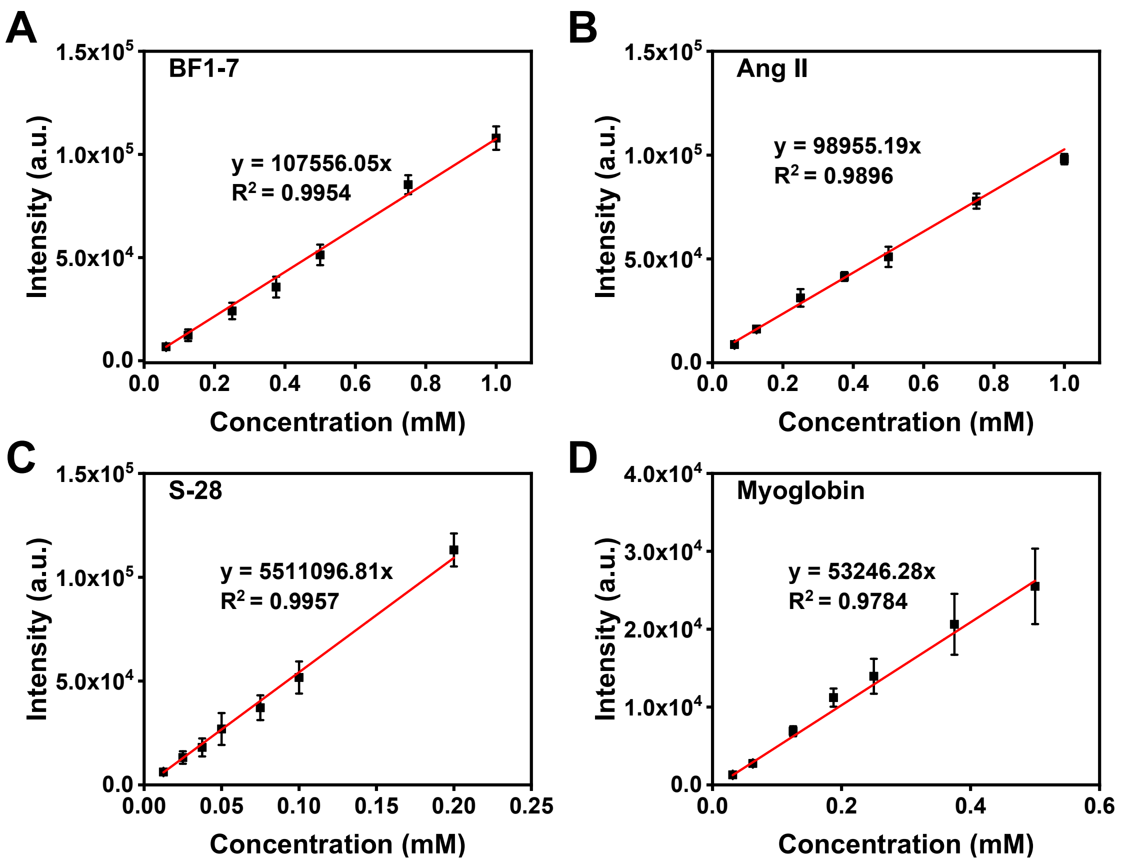

Figure S5 Standard addition curves of (A) Bradykinin Fragment 1-7, (B) Angiotensin II, (C) Somatostatin-28 and (D) Myoglobin in artificial urine samples detected by pSi-based technology. Good linearities were obtained for standard-added artificial urine samples, ranging between 0.0125 mM and 1 mM.

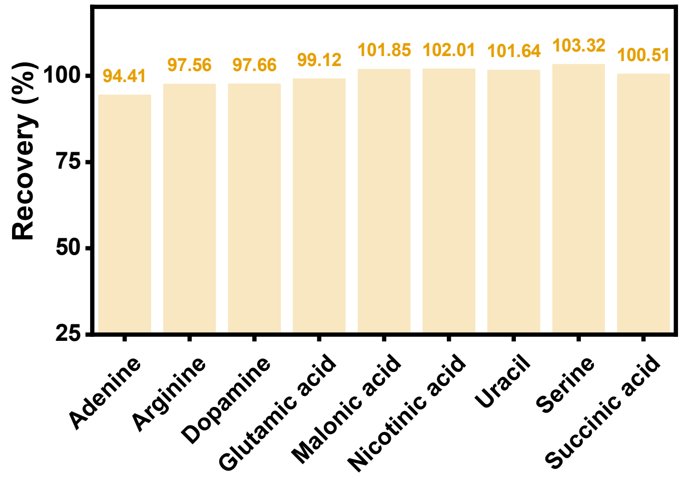

Figure S6 Recovery yield of nine metabolites (35 μM) in artificial urine samples using TCE technique.

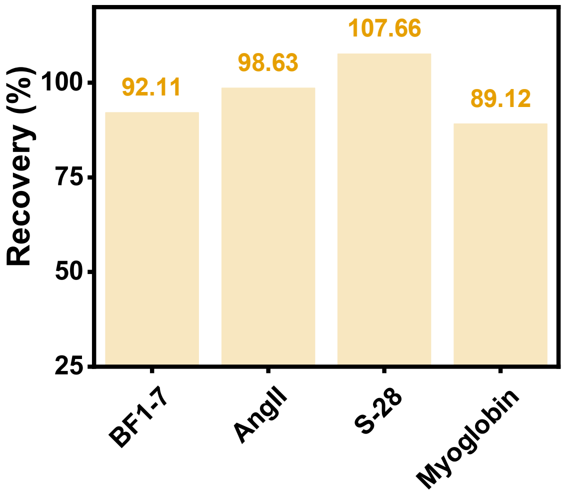

Figure S7 Recovery yield of four peptides (350 μM) in artificial urine samples using pSi-based technology.

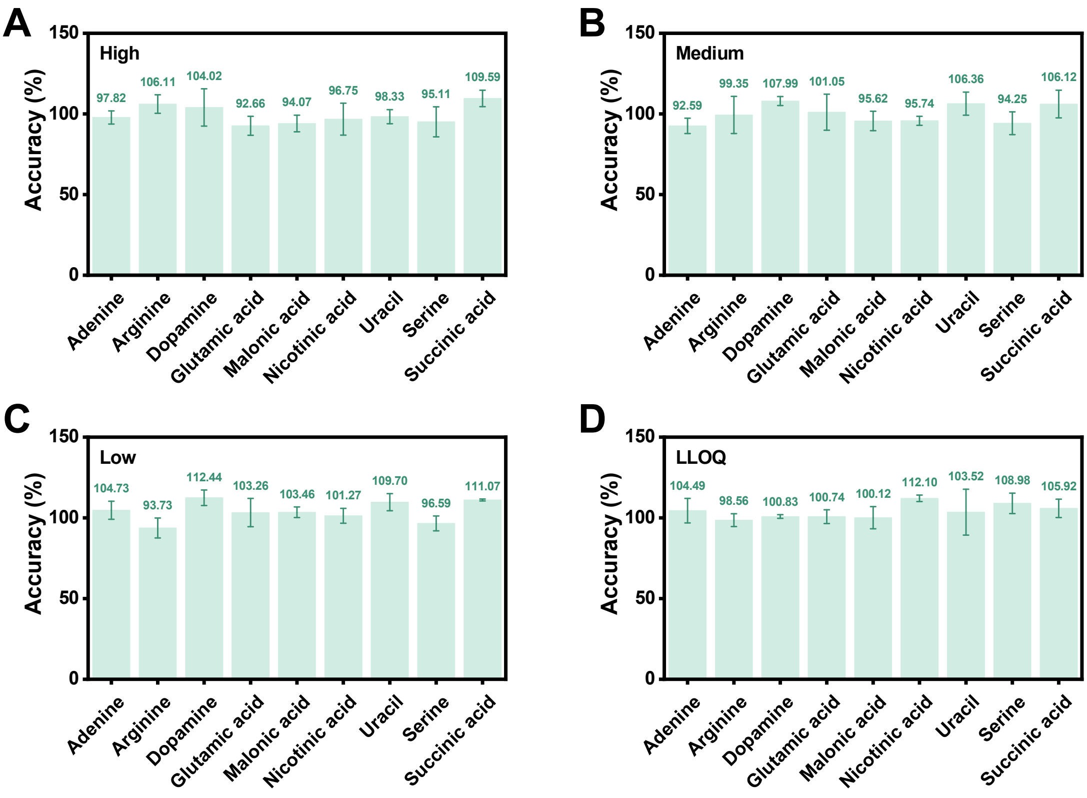

Figure S8 Accuracy of nine metabolites in artificial urine samples using TCE technique. (A) High concentration,100 μM. (B) Medium concentration, 50 μM. (C) Low concentration, 20 μM. (D) Lower limit of quantification (LLQD, 10 μM).

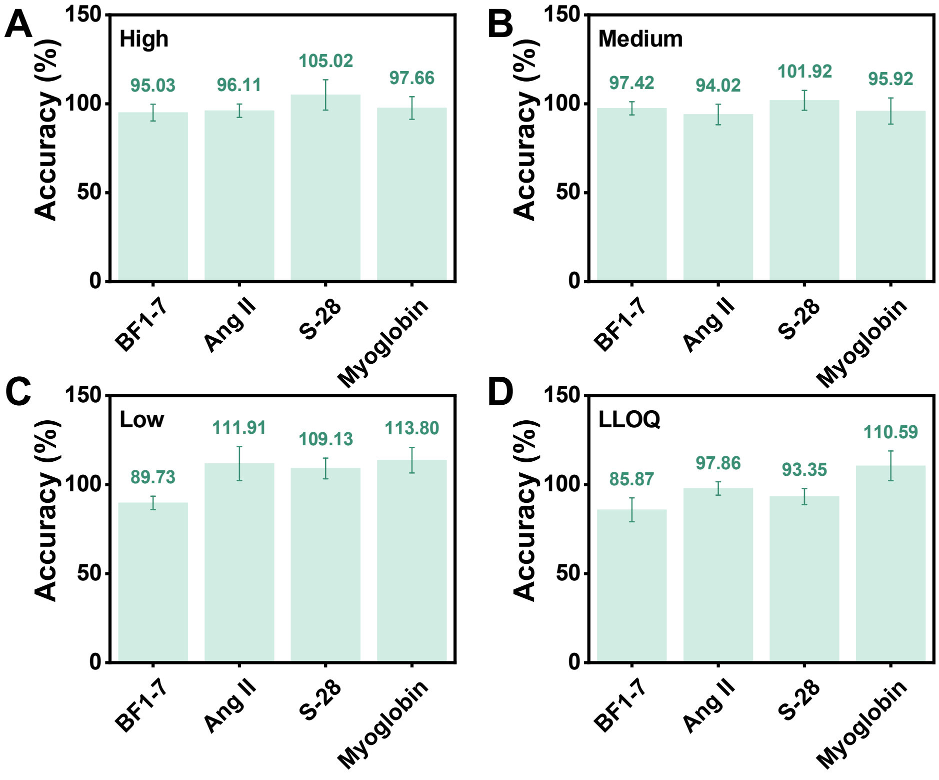

Figure S9 Accuracy of four peptides in artificial urine samples using pSi-based technology. (A) High concentration, 500 μM. (B) Medium concentration, 200 μM. (C) Low concentration, 100 μM. (D) Lower limit of quantification.

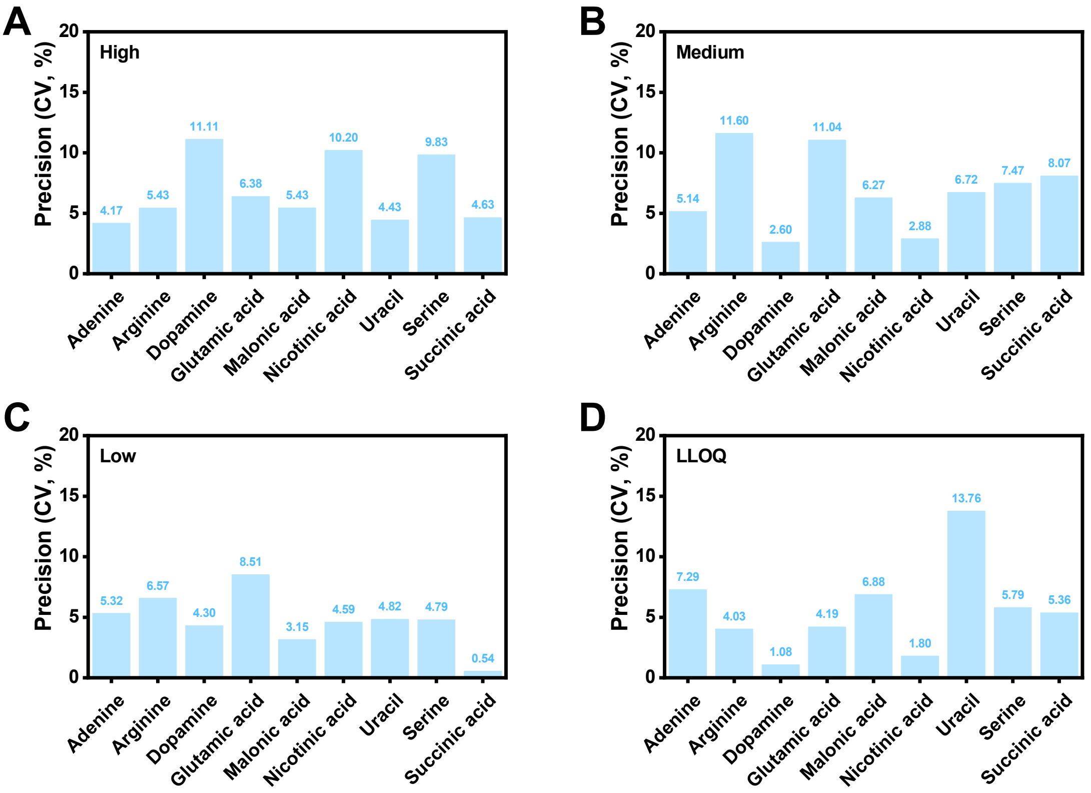

Figure S10 Precision of nine metabolites in artificial urine samples using TCE technique. (A)

High concentration,100 μM. (B) Medium concentration, 50 μM. (C) Low concentration, 20 μM. (D) Lower limit of quantification (LLQD, 10 μM).

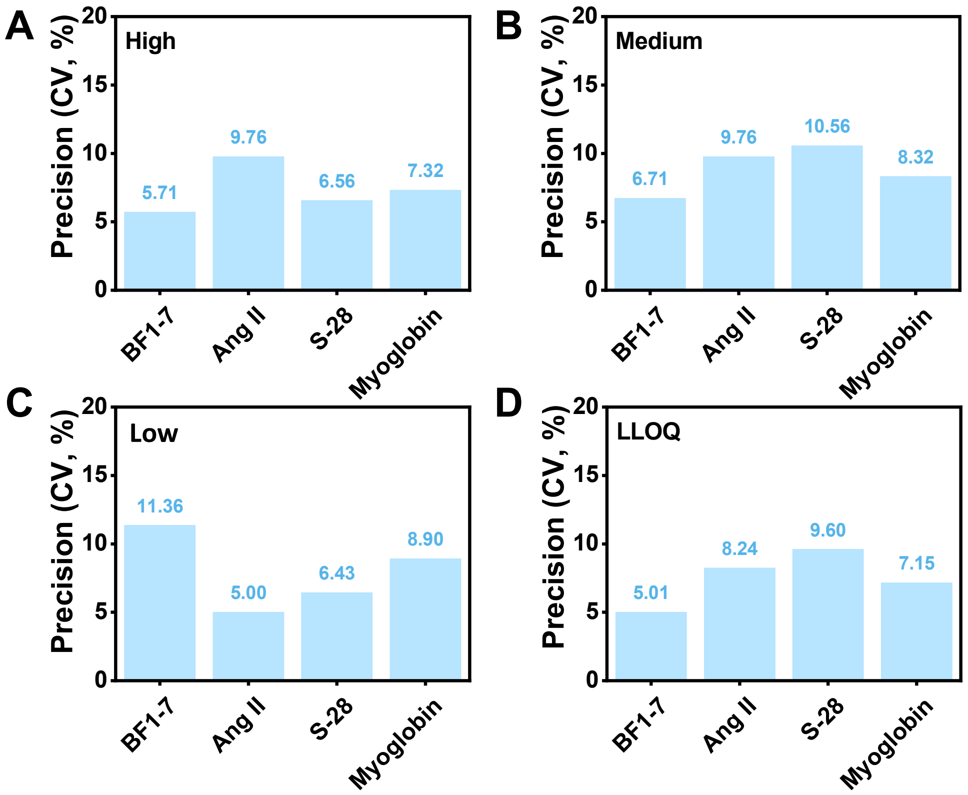

Figure S11 Precision of four peptides in artificial urine samples using pSi-based technology. (A) High concentration, 500 μM. (B) Medium concentration, 200 μM. (C) Low concentration, 100 μM. (D) Lower limit of quantification.

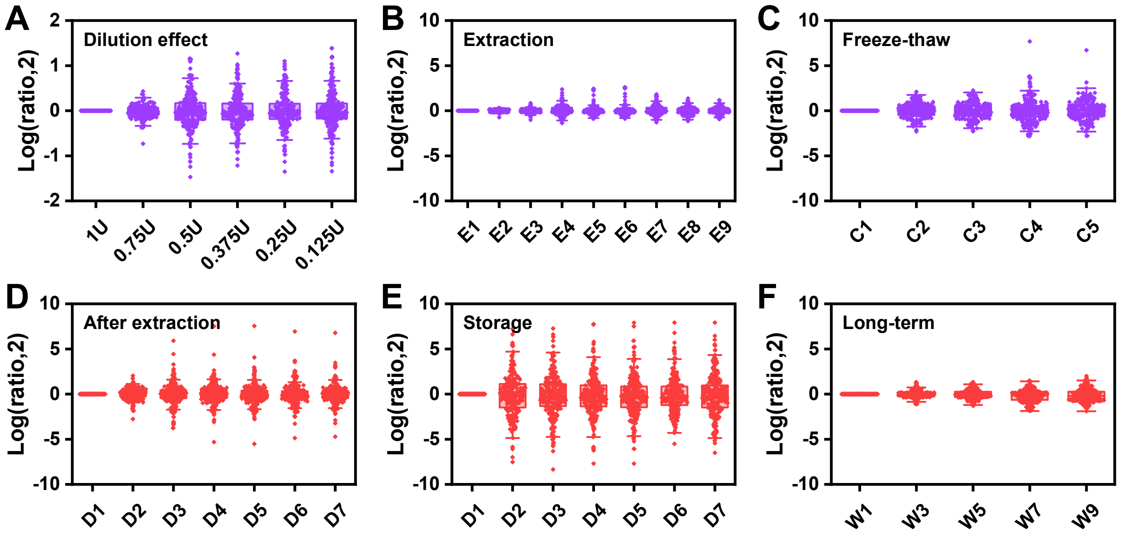

Figure S12 Dilution effect and stability of urine metabolomics. (A) Dilution correction effect was presented by calculating the peak ratios of diluted urine samples (0.75U, 0.5U, 0.375U, 0.25U,0.125U) and original 1U sample after data normalization. (B) Extraction stability was presented by calculating the peak ratios of data collected by different independent metabolite extractions. E1-E9 represent nine independent metabolite extraction experiments. (C) Freeze-thaw stability. C1-C5 represent the number of freeze-thaw cycles. (D) Long-term stability after extraction. After metabolite extraction, the chips were placed for different days until detection. D1-D7 represent the number of storage days. (E) Long-term stability before extraction. After sample pretreatment, QC urine was stored in a 4 °C refrigerator for different days until extraction. D1-D7 represent the number of storage days. (F) Long-term storage stability. QC urine samples were kept in the -80 °C refrigerator for a certain time until use. W1-W9 represent the number of storage weeks.

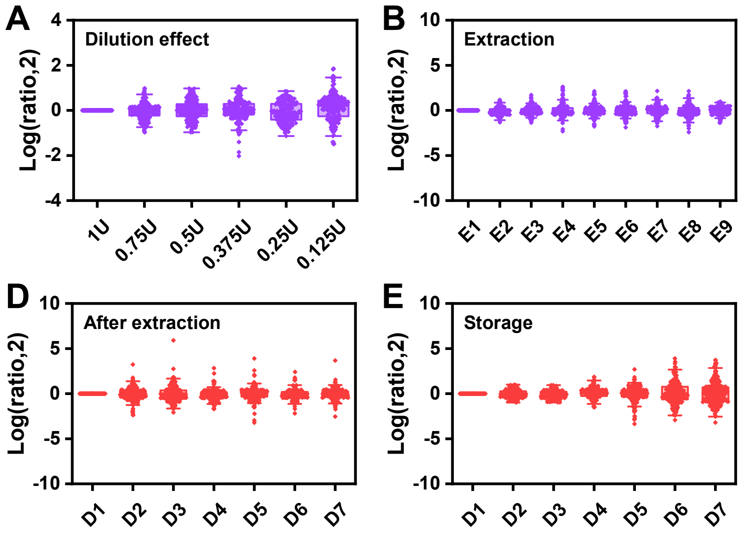

Figure S13 Dilution effect and stability of urine peptidomics. (A) Dilution correction effect between diluted urine samples and original 1U sample after data normalization. (B) Extraction stability was presented by calculating the peak ratios of data collected by different independent peptide incubation. (C) Freeze-thaw stability. (D) Long-term stability after peptide incubation. (E) Long-term stability before peptide incubation. (F) Long-term storage stability.

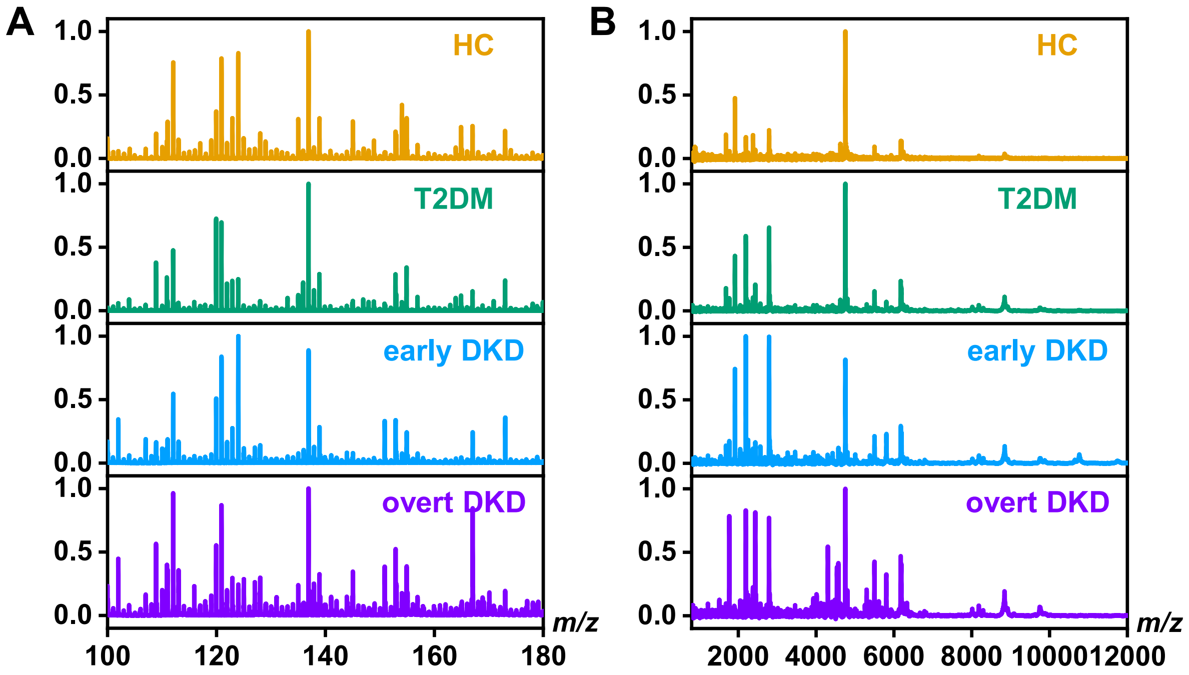

Figure S14 Representative LDI mass spectra of urine samples collected from HC, T2DM and DKD patients. (A) Metabolic fingerprint of different groups. (B) Peptide information hidden in urine samples from four groups.

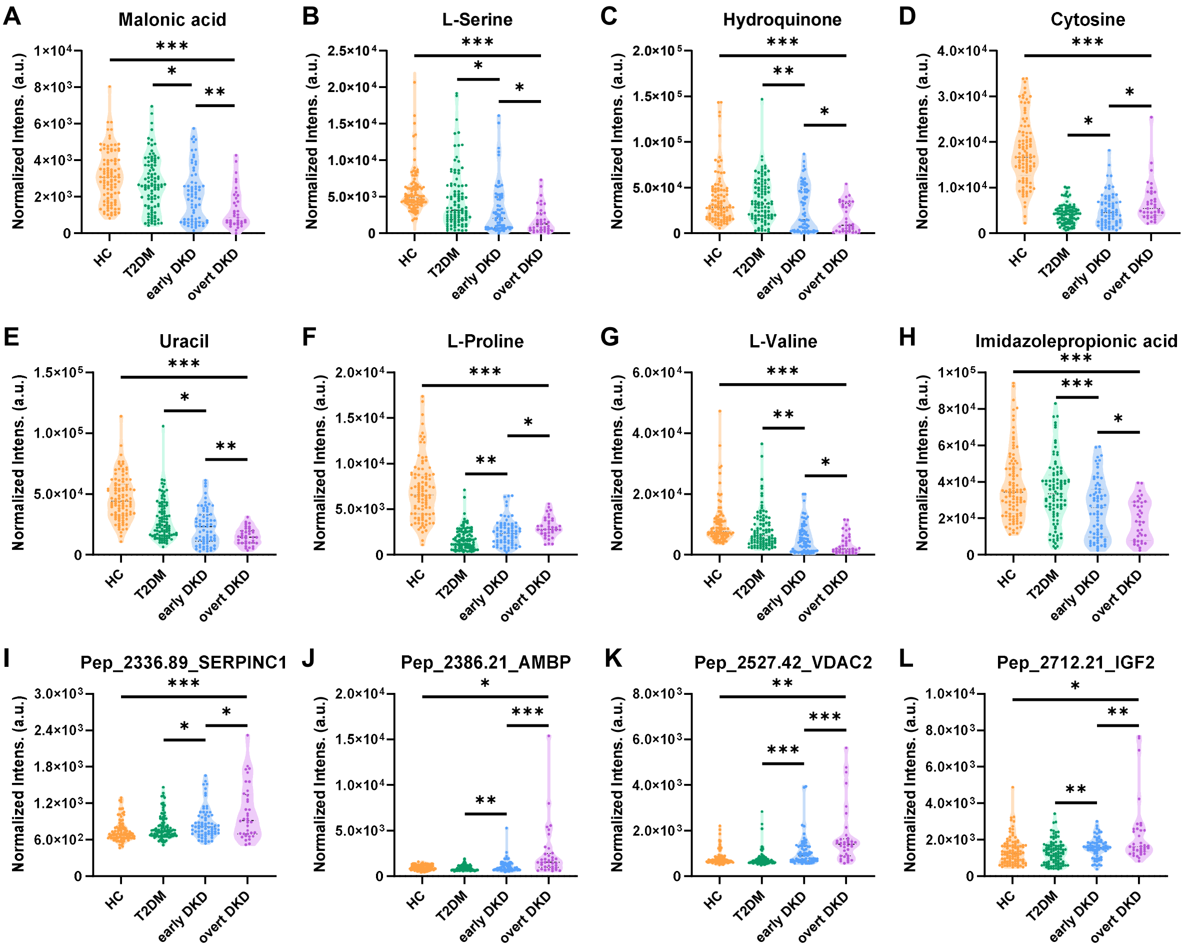

Figure S15 Box plots of the normalized intensities of stepwise differential metabolites and peptides in the HC, T2DM, early DKD and overt DKD groups (Discovery cohort). The distribution of 5-methylfuran-2-carboxylic acid, L-threonine, pep_2520.43_SERPINA1 and pep_1912.08_UMOD has been shown in the main manuscript.

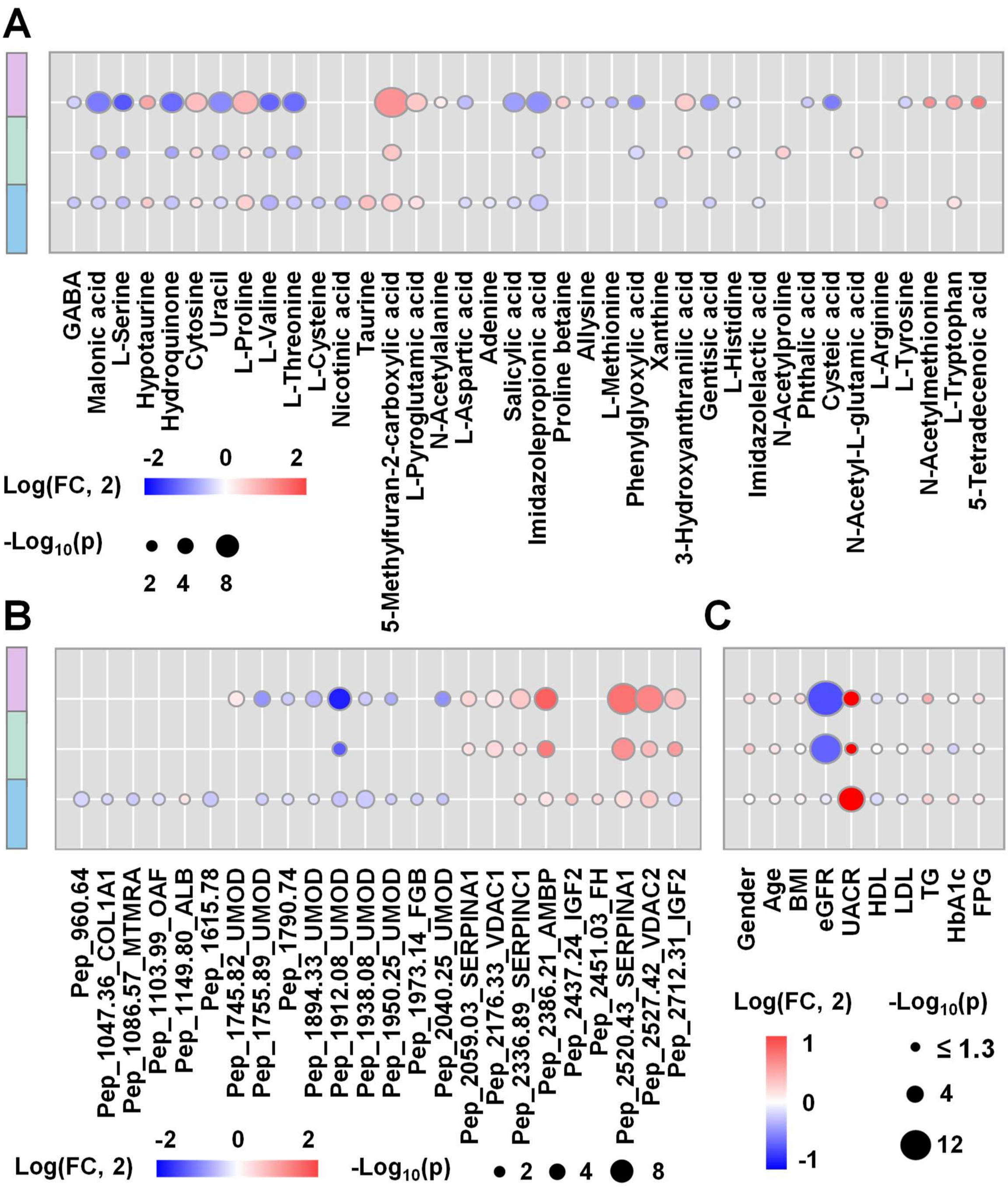

Figure S16 The circle colors represent the log2-transformed fold change of certain molecules or clinical factors between two groups. The circle size is proportional to the -log10(p) value. In the left ribbon, blue, green and purple represent pairwise analysis between T2DM and early DKD, early DKD and overt DKD, T2DM and overt DKD, respectively. (A) Metabolites. (B) Peptides. (C) Clinical factors.

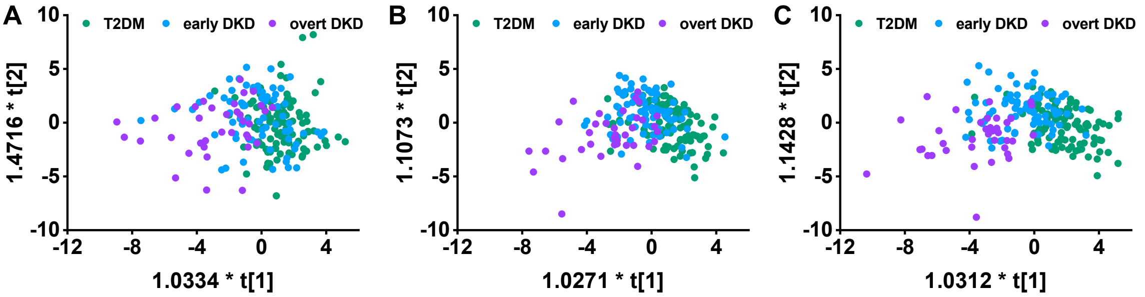

Figure S17 The OPLS-DA result for discrimination between T2DM, early DKD and overt DKD. (A) Score plot based on differential metabolites. (B) Score plot based on dysregulated peptides. (C) Score plot based on combined signatures.

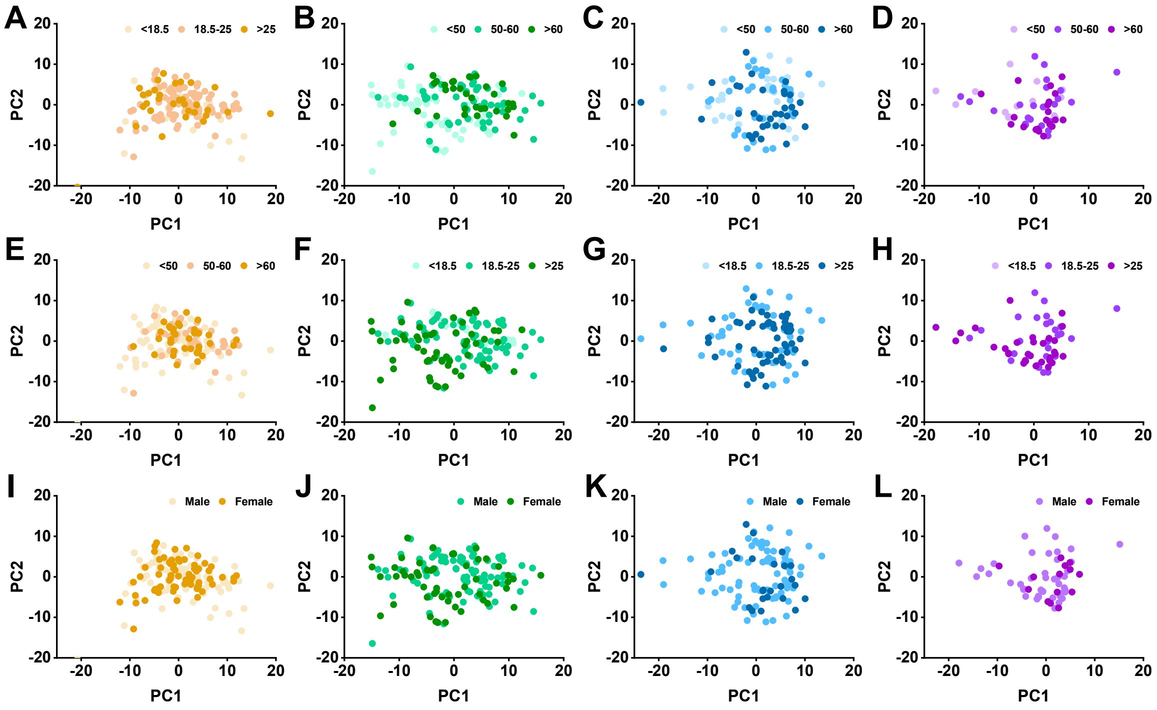

Figure S18 The PCA score plot for metabolic discrimination of individuals with different age, BMI, and gender between different subgroups. (A-D) were PCA score plots of individuals with age < 50 years old and > 50 years old in HC, T2DM, early DKD, and overt DKD group, respectively. (E-H) were PCA score plots of subjects with BMI < 18.5 kg/m^2, 18.5 ≤ BMI ≤ 25 kg/m^2, BMI > 25 kg/m^2 in four groups. (I-L) were PCA score plots of female and male subjects in four groups.

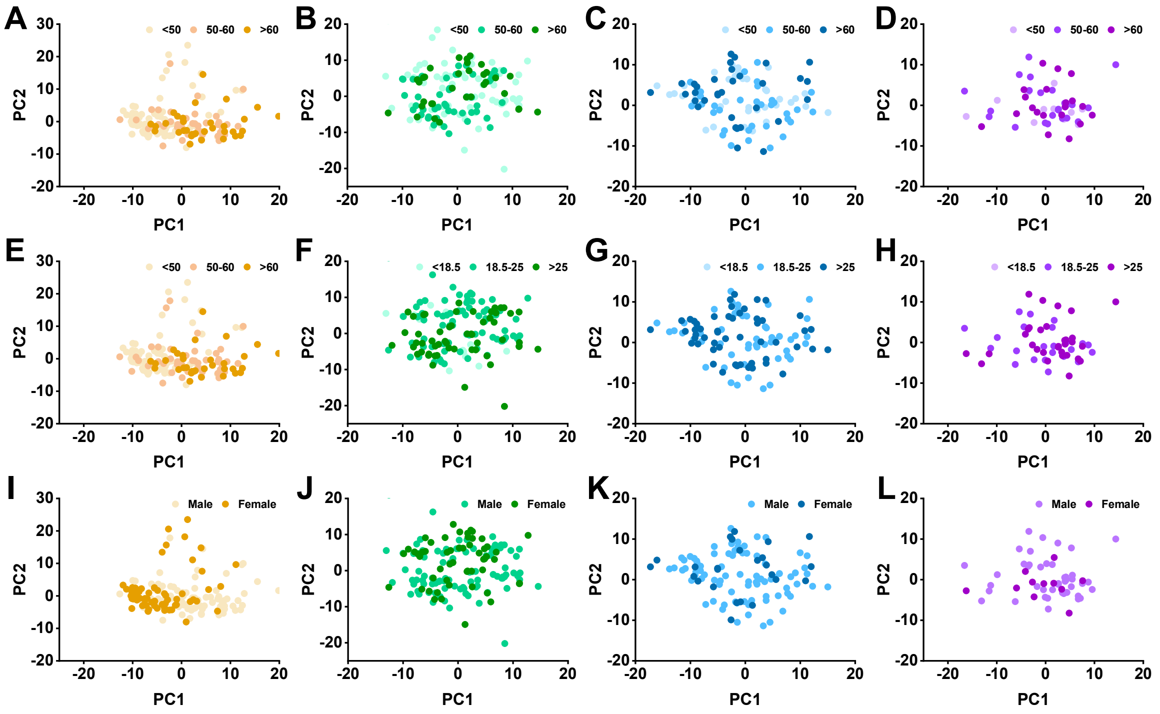

Figure S19 The PCA score plot for peptide distinction of individuals with different age, BMI, and gender between different subgroups. (A-D) were PCA score plots of individuals with age < 50 years old and > 50 years old in HC, T2DM, early DKD, and overt DKD group, respectively. (E-H) were PCA score plots of subjects with BMI < 18.5 kg/m^2, 18.5 ≤ BMI ≤ 25 kg/m^2, BMI > 25 kg/m^2 in four groups. (I-L) were PCA score plots of female and male subjects in four groups.

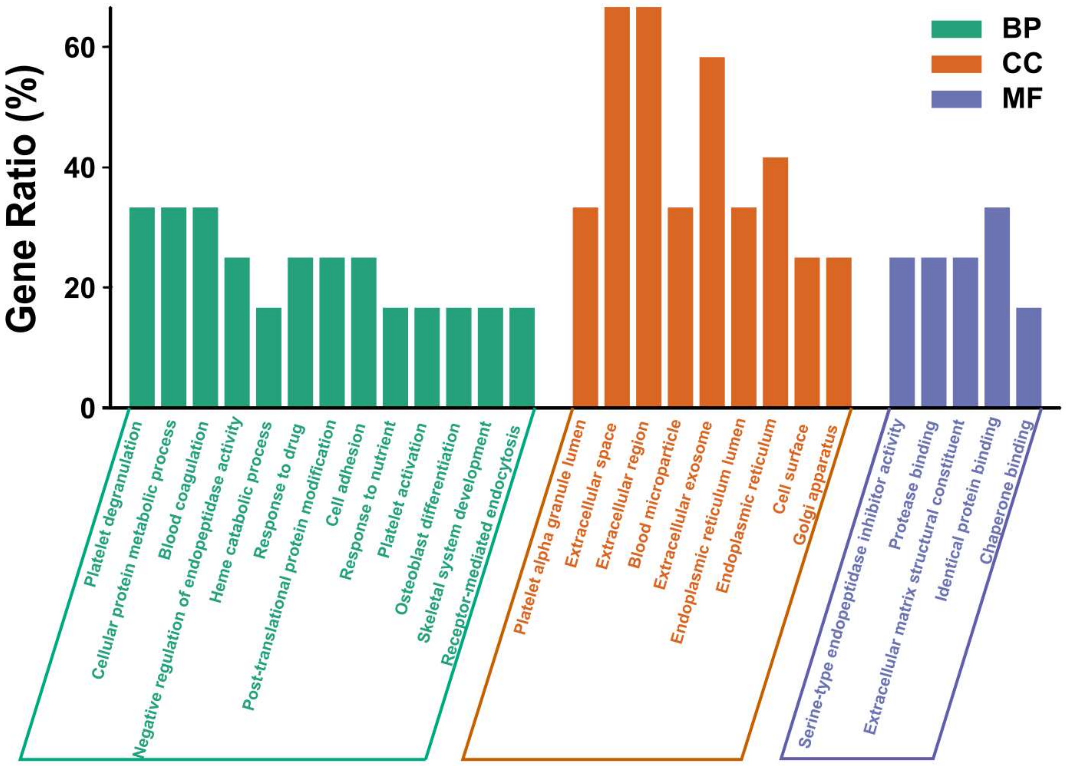

Figure S20 Functional enrichment analysis based on dysregulated peptides, including biological process (BP), cellular components (CC) and molecular function (MF).

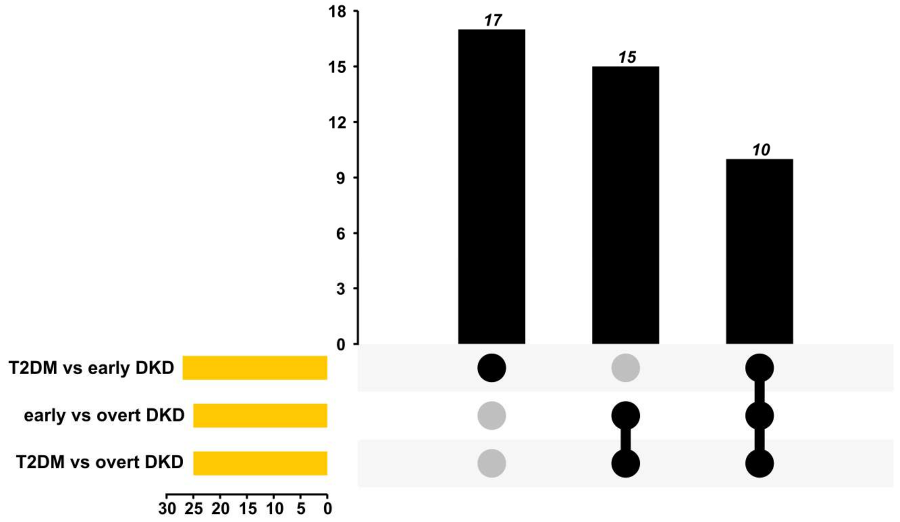

Figure S21 Intersection analysis of enriched biological processes related to DKD onset and progression. Upset plot presents the common biological processes associated with DKD development. The bar chart at the bottom left represents the number of processes included in each group. The black bar chart above represents the number of common processes in each intersection.

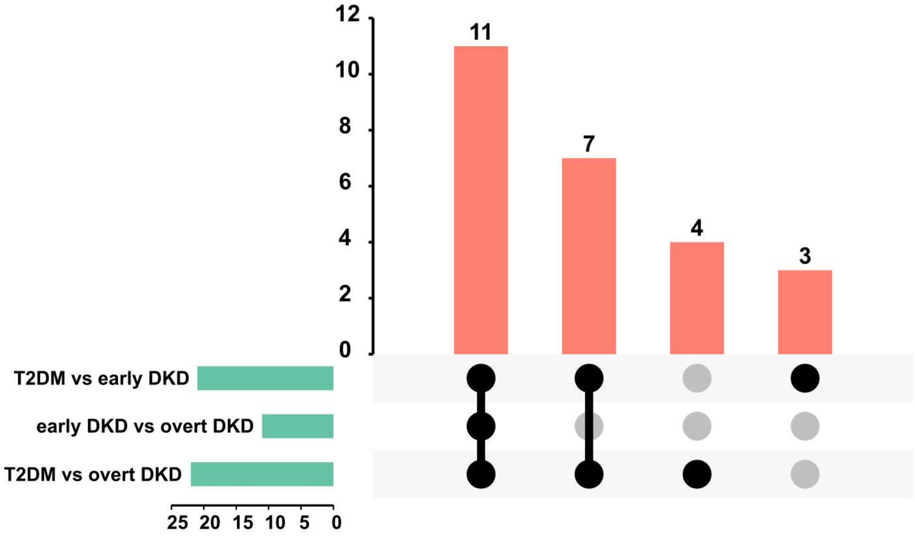

Figure S22 Intersection analysis of enriched pathways related to DKD onset and progression.

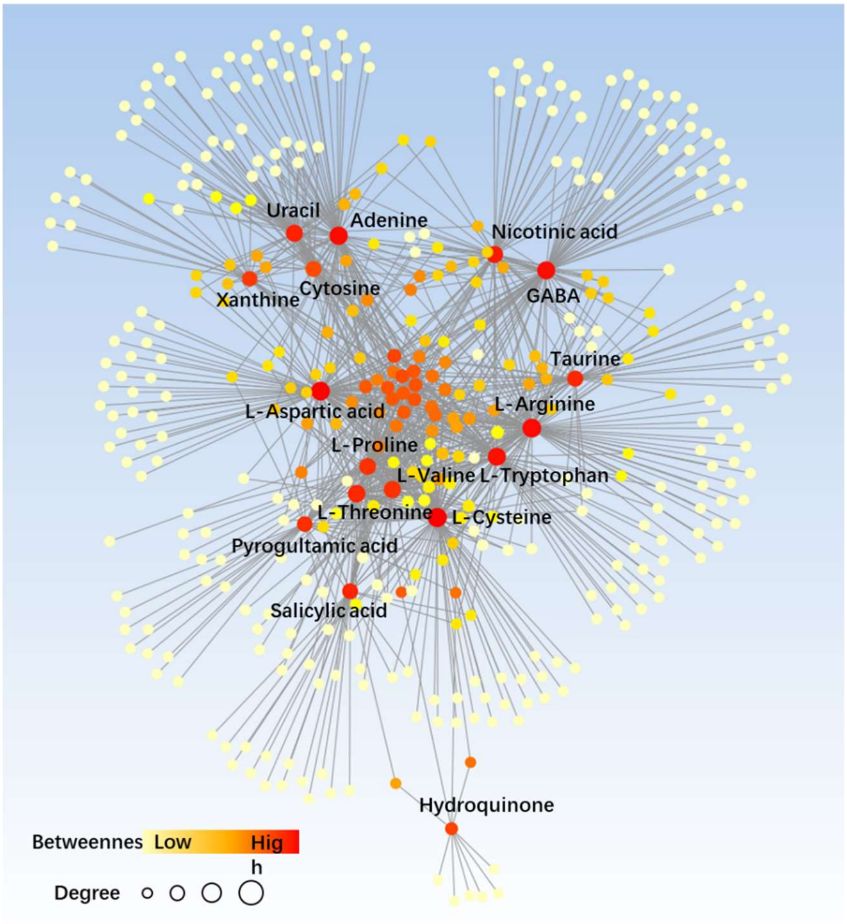

Figure S23 Metabolite-metabolite interaction network based on early DKD-related metabolites.

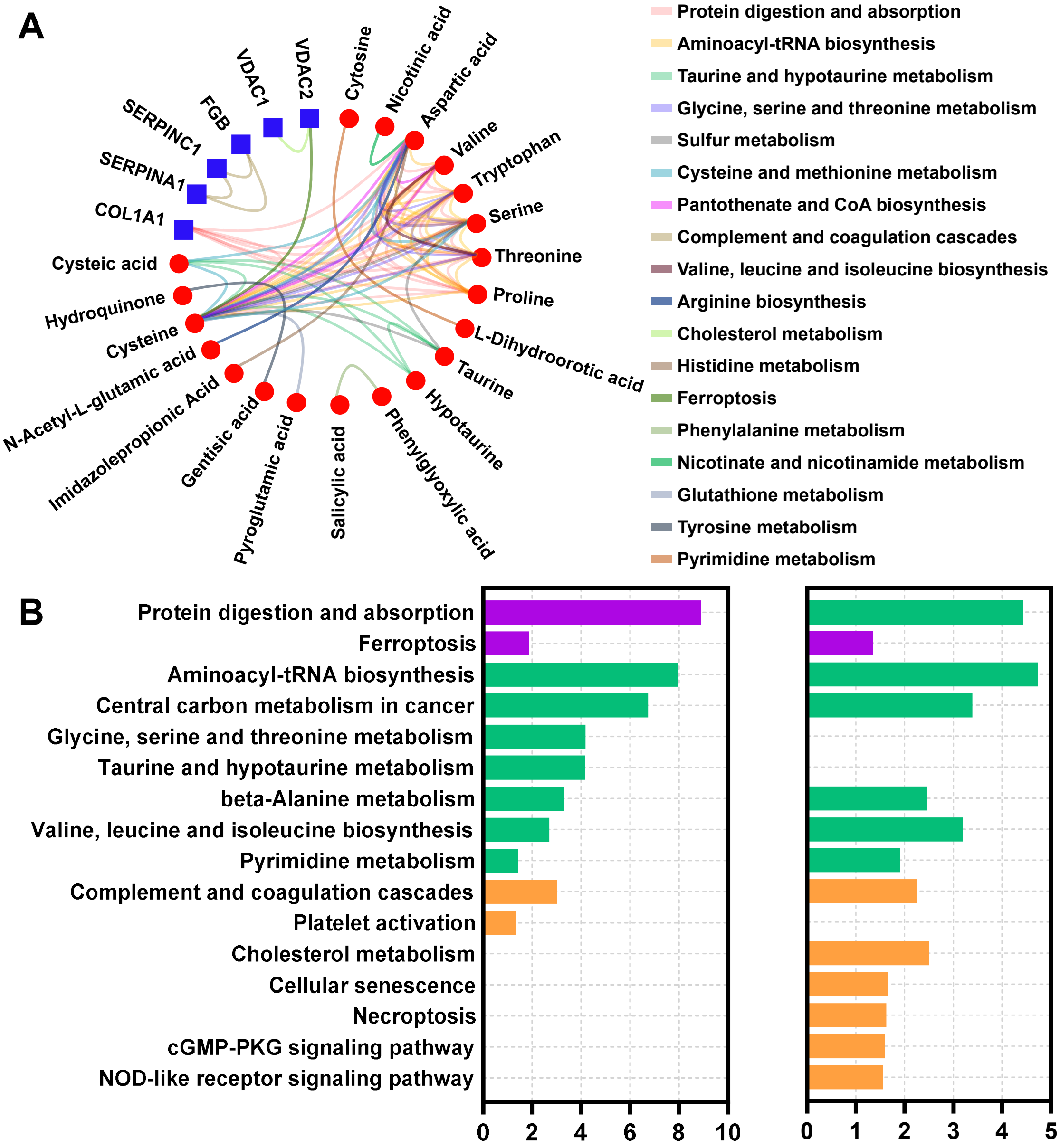

Figure S24 (A) Joint-pathway analysis associates differentially expressed metabolites with the source proteins of peptides. Peptides and metabolites are presented in blue and red nodes, respectively. (B) Pathway enrichment analysis of the network nodes reveals the top disturbed processes ($p < 0.05$) during DKD pathogenesis (left) and progression (right). The bar lengths are proportional to -log10(p) and the bar colors indicate whether the pathways were enriched in metabolites, source proteins of the peptides or their combinations.

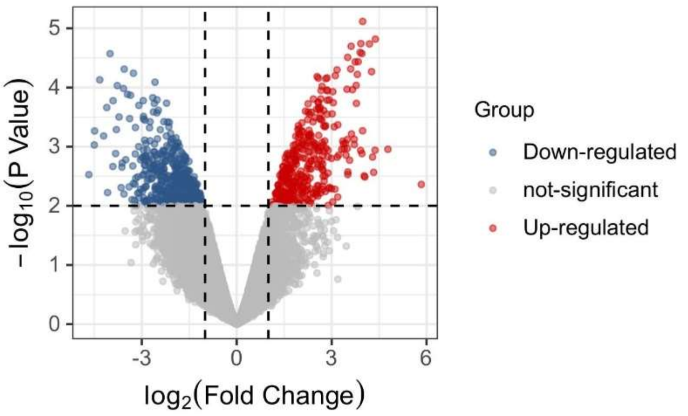

Figure S25 Volcano plot showing the differential expressed genes human kidneys with DKD and morphologically normal kidneys. Statistical significance was defined as p value < 0.01 and |$\log_2$ FC| > 1.

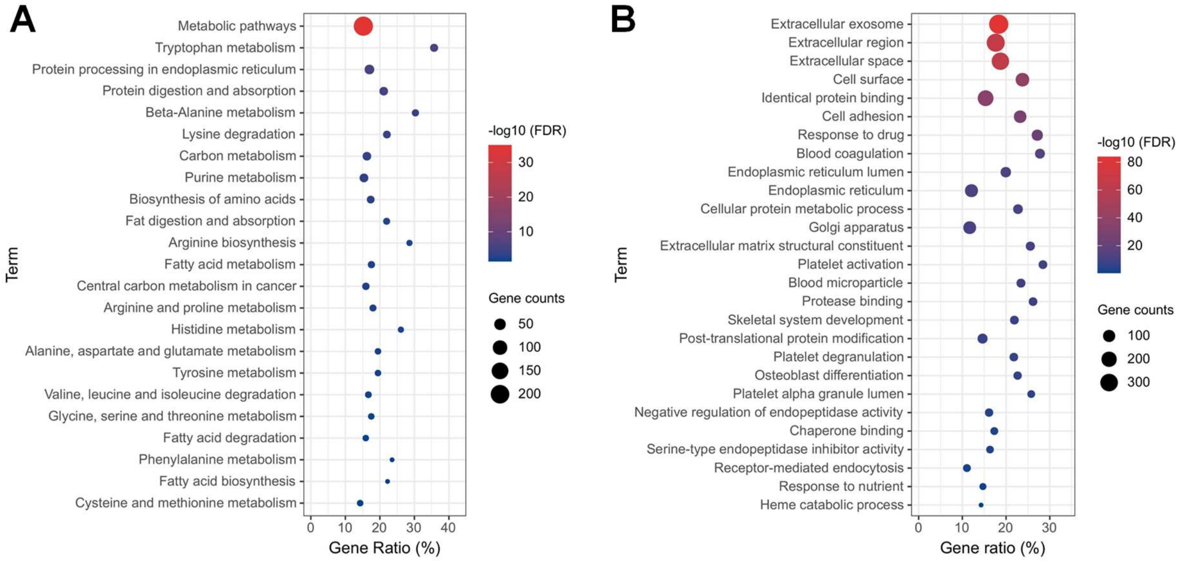

Figure S26 Gene ontology and KEGG pathway enrichment analysis of differential genes screened out by edgeR package. (A) Bubble plot of disturbed metabolic pathways related to DKD phenotype based on KEGG pathway analysis. (B) Bubble plot of gene ontology analysis.

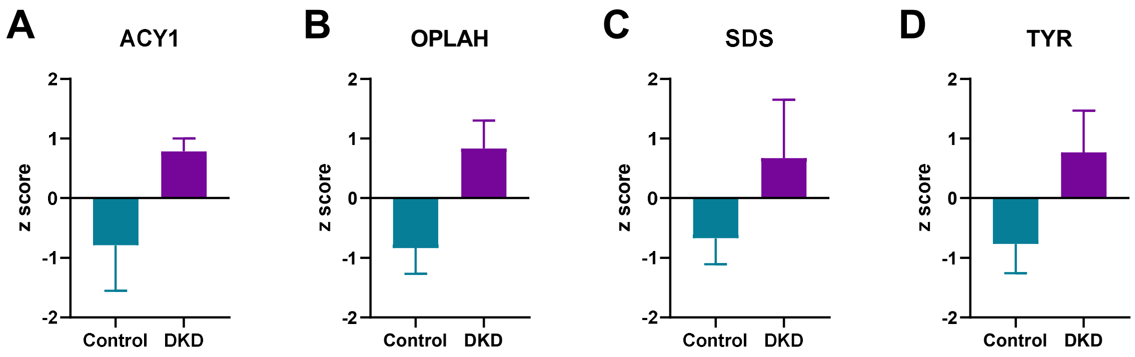

Figure S27 The expression of candidate genes in human kidneys with DKD and morphologically normal kidneys from open database (GSE1009).

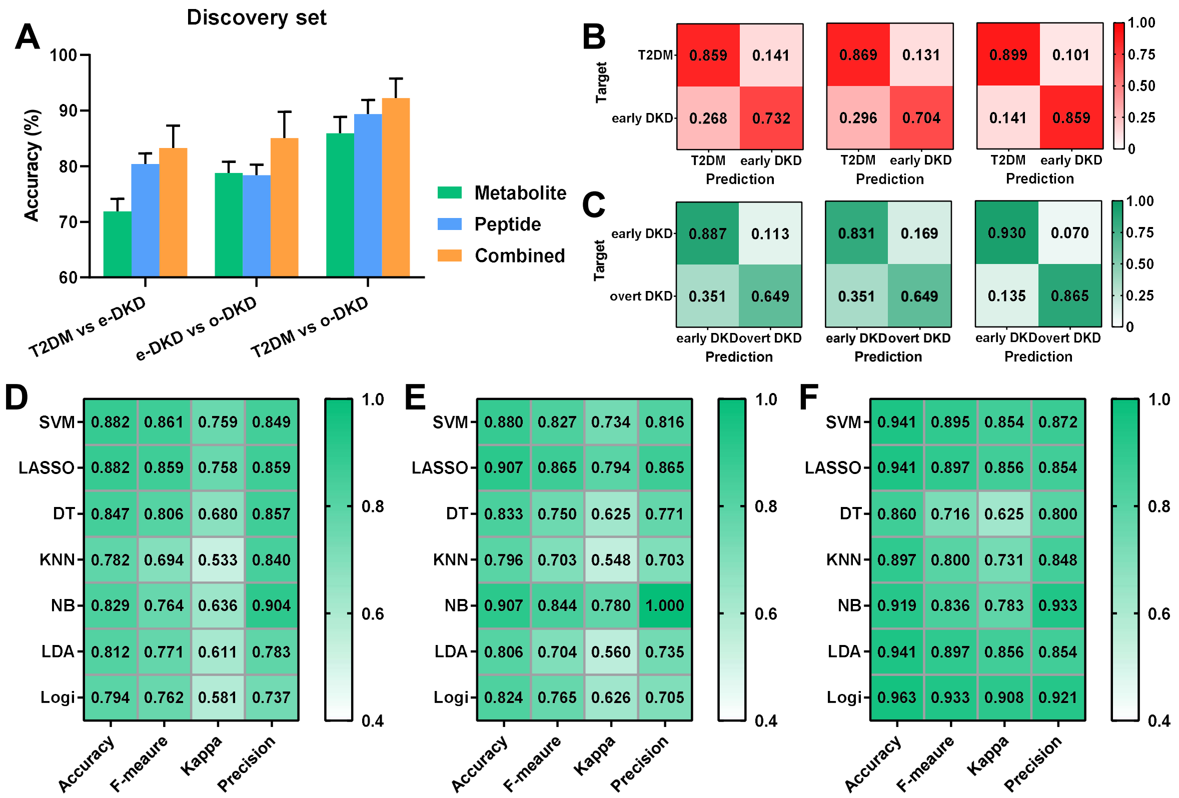

Figure S28 Pairwise analysis based on different machine learning methods in the discovery cohort. (A) General prediction accuracy based on metabolite features only (green), peptide features only (blue), and integration of peptide and metabolite features (orange). (B, C) Heatmap for the predicted ratios of early DKD diagnosis and DKD status discrimination, respectively. (D-F) Performance of different machine learning algorithms for pairwise prediction, evaluated by Kappa statistic, accuracy, F-measure, and precision. (D) T2DM vs early DKD (E) early DKD vs overt DKD (F) T2DM vs overt DKD.

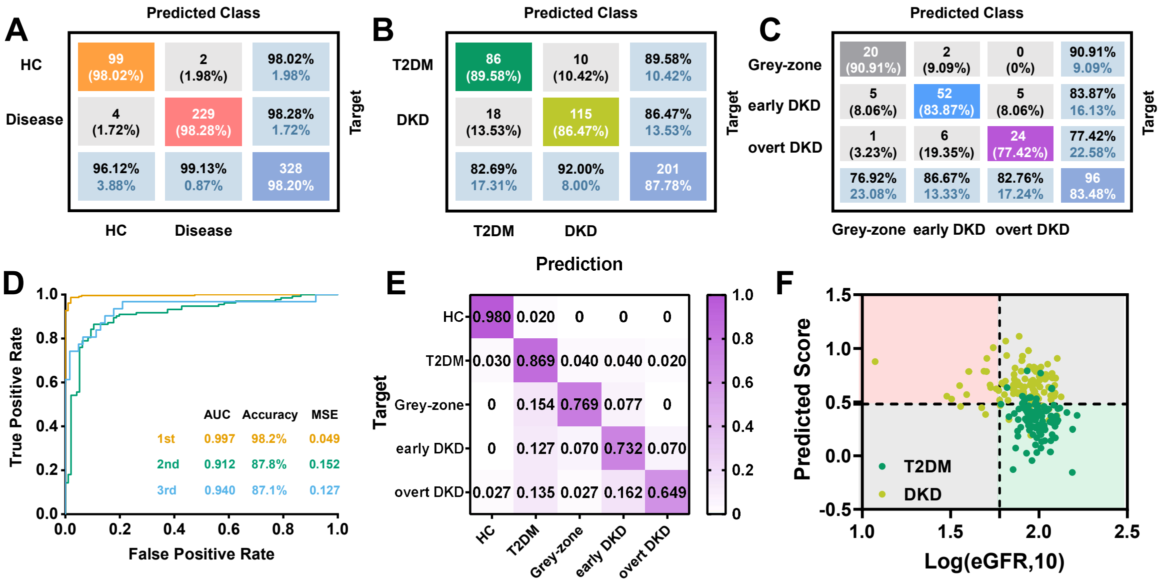

Figure S29 Diagnostic performance of the stepwise prediction model in the discovery cohort. For the discovery set, the confusion matrix and ROC curves for DKD diagnosis and DKD status prediction were shown in (A), (B) and (C), respectively. (D) Receiver operating curves for the stepwise prediction in the discovery set. (E) Heatmap for the predicted ratios of predicted cases to true cases. (F) 2D coordinate plot visualizes diagnostic results for each sample based on eGFR levels and established model.

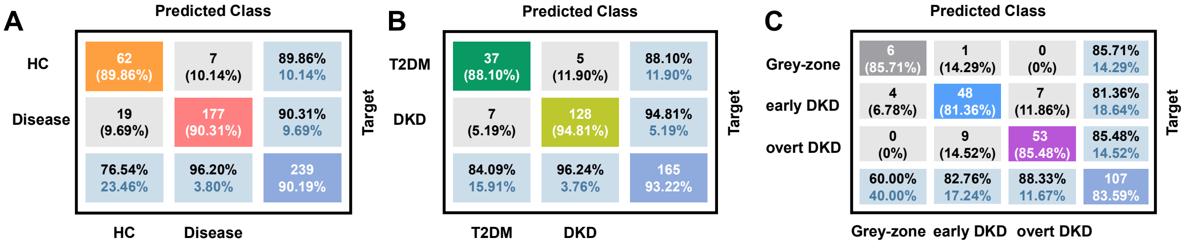

Figure S30 Confusion matrix for the (A) first, (B) second, and (C) third step diagnosis in the external validation set, respectively.

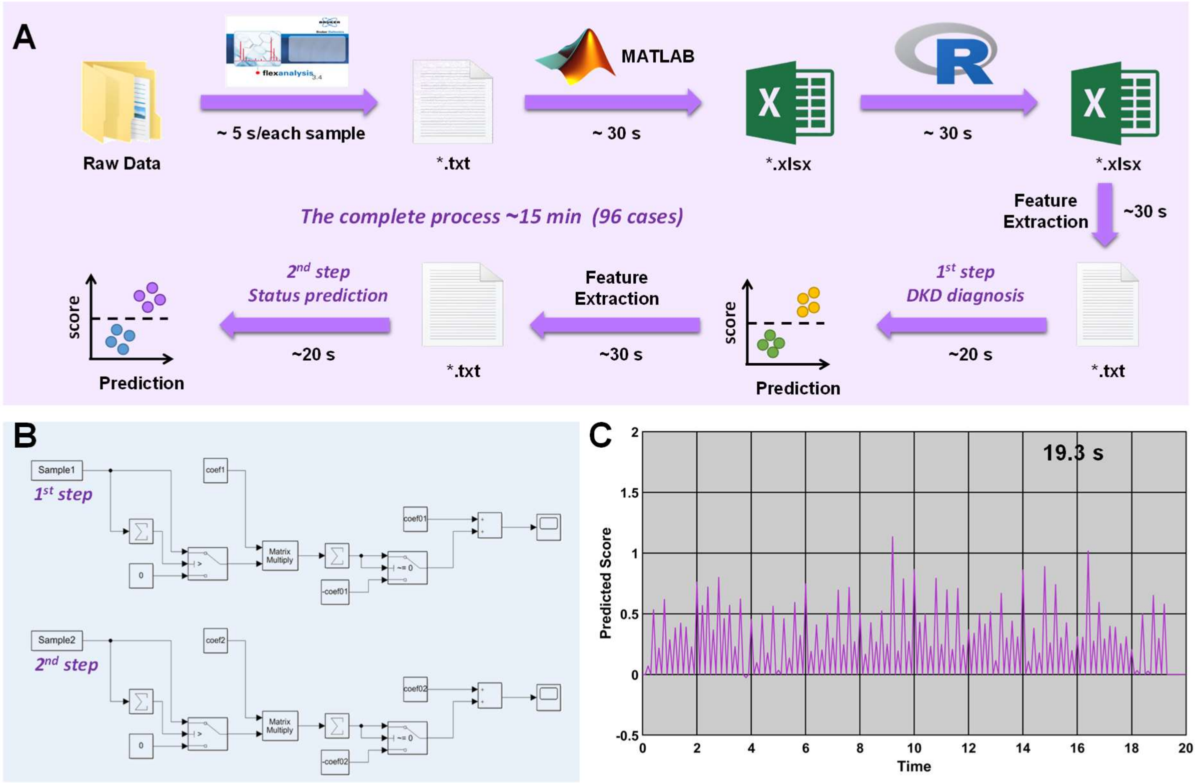

Figure S31 Pipeline of nearly real-time molecular diagnosis by multi-omics platform using the established model. (A) The pipeline for data processing includes file conversion, peak read, normalization, feature extraction and prediction. (B) The stepwise LASSO model was constructed with function blocks in Simulink. (C) The simulated real-time diagnosis process for DKD status prediction. The simulation batch used here consists of 96 cases.

3. Supplementary Tables

Table S1. Clinical characteristics of the subjects in this study.

Characteristics	Discovery set					Validation set					External validation set				
	HC	T2DM	Early DKD	Overt DKD	Grey-zone	HC	T2DM	Early DKD	Overt DKD	Grey-zone	HC	T2DM	Early DKD	Overt DKD	Grey-zone
**Cases**	101	99	71	37	26	51	50	35	18	13	69	49	69	70	8
**Men, n (%)**	56 (55%)	67 (68%)	51 (72%)	28 (76%)	20 (77%)	30 (59%)	28 (56%)	29 (83%)	15 (83%)	9 (69%)	40 (58%)	25 (51%)	51 (74%)	45 (64%)	6 (75%)
**Age (median/range)**	49.34 (29-78)	51.53 (23-85)	53.96 (24-82)	59.32 (33-81)	55.15 (41-85)	50.01 (26-78)	56.77 (32-78)	52.51 (29-75)	61.22 (46-82)	50.69 (34-69)	48.99 (24-81)	55.04 (29-80)	54.06 (29-78)	57.78 (34-88)	51.75 (24-70)
**UACR (mg/mmol)**	< 3	< 3	> 3	3-30, n=5; > 30, n=32	< 3	< 3	< 3	> 3	< 3, n=1; 3-30, n=4; > 30, n=13	< 3	< 3	< 3	> 3	< 3, n=6; 3-30, n=6; > 30, n=58	< 3
**eGFR (ml/min/1.73 m²)**	Negative	100.05 (19.04)	94.56 (18.23)	61.91 (24.05)	86.23 (10.76)	Negativ e	91. (14.76)	90.72 (15.42)	56.93 (30.07)	102.05 (9.60)	Negative	95.08 (13.72)	95.28 (15.16)	76.19 (29.13)	99.18 (12.99)
**BMI (kg/m^2)**	24.85 (2.69)	24.76 (3.07)	25.91 (3.34)	25.65 (2.92)	26.72 (3.08)	24.41 (3.07)	24.34 (2.43)	25.17 (3.57)	25.17 (3.57)	26.89 (4.05)	24.37 (2.97)	24.41 (3.49)	25.20 (3.43)	26.01 (3.21)	24.14 (1.46)
**HbA1c (%)**	n/a	8.11 (2.22)	8.01 (1.65)	8.22 (2.12)	7.83 (1.70)	n/a	7.22 (1.79)	7.51 (1.83)	8.26 (2.24)	7.77 (1.58)	n/a	6.93 (2.47)	6.99 (3.51)	7.95 (1.69)	7.58 (1.20)
**HDL (mmol/L)**	1.14 (0.33)	1.19 (0.31)	1.08 (0.25)	1.08 (0.21)	1.26 (0.59)	1.22 (0.29)	1.20 (0.28)	1.24 (0.31)	1.13 (0.43)	1.10 (0.29)	1.20 (0.45)	1.26 (0.28)	1.23 (0.28)	1.22 (0.33)	1.10 (0.22)
**LDL (mmol/L)**	2.60 (0.86)	2.68 (0.94)	2.52 (0.93)	2.55 (1.14)	2.82 (0.80)	2.49 (1.22)	2.75 (1.07)	2.63 (0.80)	2.74 (1.34)	2.40 (0.93)	2.58 (1.01)	2.47 (0.97)	2.65 (0.84)	2.64 (0.90)	2.55 (0.77)
**TG (mmol/L)**	1.64 (1.0 9)	1.68 (1.39)	1.96 (1.10)	1.87 (0.75)	1.98 (1.70)	1.59 (0.99)	1.84 (1.61)	1.53 (0.69)	2.20 (2.07)	2.00 (1.88)	1.53 (0.89)	1.49 (1.10)	1.96 (0.79)	1.95 (1.17)	2.17 (1.04)
**mALB (mg/g)**	< 30	< 30	< 30, n=16; > 30, n=55	> 30	< 30	< 30	< 30	< 30, n=3; > 30, n=32	< 30, n=3; > 30, n=15	> 30	< 30	< 30	< 30, n=31; > 30, n=37	< 30, n=16; > 30, n=54	> 30

Table S2. Summary of feature peaks that could be used to distinguish early DKD patients from T2DM subjects.

No.	Detected m/z	Type	Identification	Discovery set		Validation set		Trend
				p value	q value	p value	q value	
1	102.0608	Metabolite	GABA	3.27×10^{-3}	3.09×10^{-2}	1.68×10^{-3}	9.77×10^{-3}	↓
2	102.9920	Metabolite	Malonic acid	1.56×10^{-3}	2.22×10^{-2}	9.61×10^{-3}	3.07×10^{-2}	↓
3	104.0349	Metabolite	Serine	2.35×10^{-3}	2.67×10^{-2}	1.53×10^{-2}	4.34×10^{-2}	↓
4	108.0157	Metabolite	Hypotaurine	5.26×10^{-3}	4.12×10^{-2}	1.17×10^{-2}	3.60×10^{-2}	↑
5	109.0280	Metabolite	Hydroquinone	2.95×10^{-3}	2.91×10^{-2}	2.20×10^{-4}	2.38×10^{-3}	↓
6	110.0353	Metabolite	Cytosine	4.62×10^{-3}	3.88×10^{-2}	5.82×10^{-3}	2.24×10^{-2}	↑
7	111.0217	Metabolite	Uracil	5.53×10^{-3}	4.18×10^{-2}	1.54×10^{-3}	9.44×10^{-3}	↓
8	114.0530	Metabolite	L-Proline	1.13×10^{-3}	1.82×10^{-2}	9.87×10^{-8}	5.60×10^{-6}	↑
9	116.0686	Metabolite	L-Valine	4.11×10^{-3}	3.07×10^{-2}	1.26×10^{-2}	3.83×10^{-2}	↓
10	118.0534	Metabolite	L-Threonine	1.32×10^{-7}	2.99×10^{-5}	1.03×10^{-3}	7.30×10^{-3}	↓
11	120.0128	Metabolite	L-Cysteine	1.34×10^{-4}	6.08×10^{-3}	4.06×10^{-3}	1.77×10^{-2}	↓
12	122.0261	Metabolite	Nicotinic acid	1.77×10^{-4}	6.68×10^{-3}	1.51×10^{-2}	4.33×10^{-2}	↓
13	124.0010	Metabolite	Taurine	8.95×10^{-4}	1.69×10^{-2}	1.08×10^{-2}	3.34×10^{-2}	↑
14	125.0277	Metabolite	5-Methylfuran-2-carboxylic acid	2.50×10^{-4}	8.09×10^{-3}	1.46×10^{-3}	9.21×10^{-3}	↑
15	128.0347	Metabolite	L-Pyroglutamic acid	4.23×10^{-4}	1.20×10^{-2}	4.50×10^{-11}	1.02×10^{-8}	↑
16	132.0221	Metabolite	L-Aspartic acid	1.33×10^{-3}	2.02×10^{-2}	1.94×10^{-4}	2.20×10^{-3}	↓
17	134.0551	Metabolite	Adenine	6.30×10^{-4}	1.43×10^{-2}	6.70×10^{-4}	5.43×10^{-3}	↓
18	137.0246	Metabolite	Salicylic acid	2.11×10^{-3}	2.52×10^{-2}	1.64×10^{-2}	4.48×10^{-2}	↓

**19**	139.0515	Metabolite	Imidazolepropionic acid	4.38×10^{-3}	3.83×10^{-2}	2.72×10^{-4}	2.68×10^{-3}	↓
**20**	151.0328	Metabolite	Xanthine	7.16×10^{-5}	4.06×10^{-3}	1.47×10^{-2}	4.27×10^{-2}	↓
**21**	153.0201	Metabolite	Gentisic acid	4.91×10^{-4}	1.06×10^{-3}	2.97×10^{-3}	1.40×10^{-2}	↓
**22**	155.0451	Metabolite	Imidazolelactic acid	4.87×10^{-4}	1.23×10^{-2}	2.65×10^{-3}	1.31×10^{-2}	↓
**23**	173.1044	Metabolite	L-Arginine	2.76×10^{-3}	2.85×10^{-2}	4.59×10^{-3}	1.90×10^{-2}	↑
**24**	203.0927	Metabolite	L-Tryptophan	1.77×10^{-3}	2.36×10^{-2}	7.60×10^{-3}	2.58×10^{-2}	↑
**25**	960.64	Peptide	Pep_960.64	3.59×10^{-3}	1.89×10^{-2}	1.24×10^{-3}	9.44×10^{-3}	↓
**26**	1047.36	Peptide	Pep_1047.36_COL1A1	1.43×10^{-2}	4.46×10^{-2}	6.93×10^{-4}	6.79×10^{-3}	↓
**27**	1086.57	Peptide	Pep_1086.57_MTMRA	5.60×10^{-4}	6.41×10^{-3}	9.79×10^{-3}	4.03×10^{-2}	↓
**28**	1103.99	Peptide	Pep_1103.99_OAF	8.11×10^{-4}	7.60×10^{-3}	2.06×10^{-3}	1.28×10^{-2}	↓
**29**	1149.80	Peptide	Pep_1149.80_ALB	5.98×10^{-3}	2.68×10^{-2}	4.86×10^{-3}	2.44×10^{-2}	↑
**30**	1615.78	Peptide	Pep_1615.78	2.95×10^{-4}	4.67×10^{-3}	4.31×10^{-3}	2.28×10^{-2}	↓
**31**	1755.89	Peptide	Pep_1755.89_UMOD	1.40×10^{-2}	4.45×10^{-2}	6.33×10^{-5}	1.00×10^{-3}	↓
**32**	1790.74	Peptide	Pep_1790.74	6.67×10^{-3}	2.80×10^{-2}	5.13×10^{-3}	2.52×10^{-2}	↓
**33**	1894.33	Peptide	Pep_1894.33_UMOD	1.06×10^{-2}	3.76×10^{-2}	6.90×10^{-7}	2.03×10^{-5}	↓
**34**	1912.08	Peptide	Pep_1912.08_UMOD	3.96×10^{-3}	1.99×10^{-2}	9.00×10^{-9}	6.18×10^{-7}	↓
**35**	1938.08	Peptide	Pep_1938.08_UMOD	3.04×10^{-5}	1.04×10^{-3}	3.42×10^{-6}	7.83×10^{-5}	↓
**36**	1950.25	Peptide	Pep_1950.25_UMOD	1.19×10^{-2}	4.09×10^{-2}	2.40×10^{-9}	4.94×10^{-7}	↓
**37**	1973.14	Peptide	Pep_1973.14_FGB	1.52×10^{-3}	1.12×10^{-2}	1.76×10^{-3}	1.17×10^{-2}	↓
**38**	2040.25	Peptide	Pep_2040.25_UMOD	7.58×10^{-4}	7.44×10^{-3}	2.37×10^{-8}	1.22×10^{-6}	↓

**39**	2336.89	Peptide	Pep_2336.89_SERPINC1	8.53×10^{-4}	7.64×10^{-3}	1.08×10^{-2}	4.37×10^{-2}	↑
**40**	2386.21	Peptide	Pep_2386.21_AMBP	1.68×10^{-3}	1.15×10^{-2}	5.16×10^{-4}	5.59×10^{-3}	↑
**41**	2437.24	Peptide	Pep_2437.24_IGF2	3.34×10^{-3}	1.81×10^{-2}	3.11×10^{-3}	1.78×10^{-2}	↑
**42**	2451.03	Peptide	Pep_2451.03_FH	5.21×10^{-3}	2.44×10^{-2}	3.69×10^{-3}	1.99×10^{-2}	↓
**43**	2520.43	Peptide	Pep_2520.43_SERPINA1	1.25×10^{-3}	9.51×10^{-3}	2.58×10^{-4}	2.96×10^{-3}	↑
**44**	2527.42	Peptide	Pep_2527.42_VDAC2	5.44×10^{-3}	2.49×10^{-2}	9.40×10^{-4}	8.07×10^{-3}	↑
**45**	2712.31	Peptide	Pep_2712.31_IGF2	1.28×10^{-2}	4.26×10^{-2}	1.81×10^{-4}	2.19×10^{-3}	↑

Table S3. Summary of feature peaks that could be used to distinguish early DKD patients from overt DKD subjects.

No.	Detected m/z	Type	Identification	Discovery set		Validation set		Trend
				p value	q value	p value	q value	
**1**	102.9920	Metabolite	Malonic acid	8.42×10^{-6}	4.78×10^{-4}	1.26×10^{-5}	4.78×10^{-4}	↓
**2**	104.0349	Metabolite	Serine	4.71×10^{-3}	2.67×10^{-2}	5.30×10^{-3}	2.67×10^{-2}	↓
**3**	109.0280	Metabolite	Hydroquinone	3.17×10^{-3}	2.25×10^{-2}	3.76×10^{-3}	2.25×10^{-2}	↓
**4**	110.0353	Metabolite	Cytosine	4.36×10^{-3}	2.54×10^{-2}	7.02×10^{-7}	7.97×10^{-5}	↑
**5**	111.0217	Metabolite	Uracil	6.47×10^{-3}	3.26×10^{-2}	1.29×10^{-3}	9.47×10^{-3}	↓
**6**	114.0530	Metabolite	L-Proline	1.25×10^{-7}	2.84×10^{-5}	3.01×10^{-6}	2.27×10^{-4}	↑
**7**	116.0686	Metabolite	L-Valine	5.45×10^{-3}	2.88×10^{-2}	4.55×10^{-3}	2.40×10^{-2}	↓
**8**	118.0534	Metabolite	L-Threonine	3.76×10^{-3}	2.37×10^{-2}	7.23×10^{-4}	5.86×10^{-3}	↓
**9**	125.0277	Metabolite	5-Methylfuran-2-carboxylic acid	3.60×10^{-4}	4.54×10^{-3}	4.00×10^{-4}	4.54×10^{-3}	↑
**10**	139.0515	Metabolite	Imidazolepropionic acid	7.00×10^{-3}	3.38×10^{-2}	3.43×10^{-3}	2.11×10^{-2}	↓

**11**	149.0332	Metabolite	Phenylglyoxylic acid	8.84×10^{-5}	1.82×10^{-3}	1.04×10^{-4}	1.82×10^{-3}	↓
**12**	152.0288	Metabolite	3-Hydroxyanthranilic acid	9.16×10^{-3}	4.24×10^{-2}	1.93×10^{-7}	4.38×10^{-5}	↑
**13**	154.0590	Metabolite	L-Histidine	7.57×10^{-5}	1.72×10^{-3}	9.09×10^{-5}	1.72×10^{-3}	↓
**14**	156.0677	Metabolite	N-Acetylproline	2.89×10^{-4}	4.10×10^{-3}	3.25×10^{-4}	4.10×10^{-3}	↑
**15**	170.0331	Metabolite	N-Acetylglutamic acid	7.57×10^{-3}	3.58×10^{-2}	5.85×10^{-4}	5.31×10^{-3}	↑
**16**	1912.08	Peptide	Pep_1912.08_UMOD	1.77×10^{-4}	3.04×10^{-3}	5.52×10^{-3}	3.55×10^{-2}	↓
**17**	2059.03	Peptide	Pep_2059.03_SERPINA1	3.26×10^{-3}	2.31×10^{-2}	2.13×10^{-4}	4.87×10^{-3}	↑
**18**	2176.33	Peptide	Pep_2176.33_VDAC1	1.47×10^{-6}	5.04×10^{-5}	1.96×10^{-5}	1.01×10^{-3}	↑
**19**	2336.89	Peptide	Pep_2336.89_SERPINC1	1.08×10^{-2}	4.82×10^{-2}	6.08×10^{-4}	8.95×10^{-3}	↑
**20**	2386.21	Peptide	Pep_2386.21_AMBP	2.00×10^{-6}	5.90×10^{-5}	8.51×10^{-3}	4.74×10^{-2}	↑
**21**	2520.43	Peptide	Pep_2520.43_SERPINA1	1.33×10^{-8}	9.13×10^{-7}	2.96×10^{-7}	6.10×10^{-5}	↑
**22**	2527.42	Peptide	Pep_2527.42_VDAC2	2.26×10^{-3}	1.79×10^{-2}	6.90×10^{-5}	2.03×10^{-3}	↑
**23**	2712.31	Peptide	Pep_2712.31_IGF2	3.66×10^{-3}	2.51×10^{-2}	7.60×10^{-3}	4.35×10^{-2}	↑

Table S4. Summary of feature peaks that could be used to distinguish overt DKD patients from T2DM subjects.

No.	Detected m/z	Type	Identification	Discovery set		Validation set		Trend
				p value	q value	p value	q value	
**1**	102.0608	Metabolite	GABA	3.64×10^{-3}	1.53×10^{-2}	6.66×10^{-3}	1.96×10^{-2}	↓
**2**	102.9920	Metabolite	Malonic acid	9.95×10^{-10}	1.13×10^{-7}	2.93×10^{-8}	1.11×10^{-6}	↓
**3**	104.0349	Metabolite	Serine	6.39×10^{-5}	8.05×10^{-4}	6.65×10^{-4}	3.51×10^{-3}	↓
**4**	108.0157	Metabolite	Hypotaurine	4.36×10^{-3}	1.77×10^{-2}	8.60×10^{-3}	2.38×10^{-2}	↑

**5**	109.0280	Metabolite	Hydroquinone	$3.49×10^{-6}$	$7.92×10^{-5}$	$2.37×10^{-6}$	$4.89×10^{-5}$	↓
**6**	110.0353	Metabolite	Cytosine	$3.22×10^{-7}$	$1.04×10^{-5}$	$2.72×10^{-13}$	$3.09×10^{-11}$	↑
**7**	111.0217	Metabolite	Uracil	$2.57×10^{-4}$	$1.88×10^{-3}$	$1.01×10^{-5}$	$1.43×10^{-4}$	↓
**8**	114.0530	Metabolite	L-Proline	$1.42×10^{-11}$	$3.23×10^{-9}$	$7.04×10^{-23}$	$1.60×10^{-20}$	↑
**9**	116.0686	Metabolite	L-Valine	$1.41×10^{-5}$	$2.46×10^{-4}$	$8.05×10^{-4}$	$4.06×10^{-3}$	↓
**10**	118.0534	Metabolite	L-Threonine	$4.22×10^{-9}$	$2.39×10^{-7}$	$3.70×10^{-5}$	$4.20×10^{-4}$	↓
**11**	125.0277	Metabolite	5-Methylfuran-2-carboxylic acid	$1.96×10^{-9}$	$1.48×10^{-7}$	$4.19×10^{-9}$	$2.38×10^{-7}$	↑
**12**	128.0347	Metabolite	L-Pyroglutamic acid	$1.88×10^{-7}$	$7.09×10^{-6}$	$2.37×10^{-3}$	$9.78×10^{-3}$	↑
**13**	130.0465	Metabolite	N-Acetylalanine	$1.47×10^{-2}$	$4.21×10^{-2}$	$9.15×10^{-3}$	$2.44×10^{-2}$	↑
**14**	132.0221	Metabolite	L-Aspartic acid	$5.46×10^{-5}$	$7.29×10^{-4}$	$9.69×10^{-5}$	$8.15×10^{-4}$	↓
**15**	137.0246	Metabolite	Salicylic acid	$1.11×10^{-4}$	$1.15×10^{-3}$	$4.43×10^{-5}$	$4.78×10^{-4}$	↓
**16**	139.0515	Metabolite	Imidazolepropionic acid	$7.80×10^{-6}$	$1.61×10^{-4}$	$2.07×10^{-8}$	$9.40×10^{-7}$	↓
**17**	142.0704	Metabolite	Proline betaine	$4.39×10^{-5}$	$6.23×10^{-4}$	$4.95×10^{-13}$	$3.74×10^{-11}$	↑
**18**	144.0626	Metabolite	Allysine	$8.03×10^{-3}$	$2.64×10^{-2}$	$2.74×10^{-3}$	$1.07×10^{-2}$	↓
**19**	148.0404	Metabolite	L-Methionine	$7.06×10^{-3}$	$2.39×10^{-2}$	$5.31×10^{-3}$	$1.68×10^{-2}$	↓
**20**	149.0332	Metabolite	Phenylglyoxylic acid	$1.58×10^{-3}$	$8.53×10^{-3}$	$5.81×10^{-5}$	$6.00×10^{-4}$	↓
**21**	152.0288	Metabolite	3-Hydroxyanthranilic acid	$1.04×10^{-3}$	$6.07×10^{-3}$	$3.15×10^{-3}$	$1.19×10^{-2}$	↑
**22**	153.0201	Metabolite	Gentisic acid	$2.19×10^{-3}$	$1.06×10^{-2}$	$7.86×10^{-6}$	$1.27×10^{-4}$	↓
**23**	154.0590	Metabolite	L-Histidine	$1.92×10^{-3}$	$9.92×10^{-3}$	$9.59×10^{-4}$	$4.54×10^{-3}$	↓
**24**	165.0147	Metabolite	Phthalic acid	$1.39×10^{-2}$	$4.04×10^{-2}$	$5.09×10^{-3}$	$1.65×10^{-2}$	↓

**25**	167.9989	Metabolite	Cysteic acid	6.88×10^{-3}	2.37×10^{-2}	6.42×10^{-3}	1.92×10^{-2}	↓
**26**	180.0696	Metabolite	L-Tyrosine	2.57×10^{-3}	1.22×10^{-2}	9.02×10^{-3}	2.44×10^{-2}	↓
**27**	190.0619	Metabolite	N-Acetylmethionine	2.69×10^{-3}	1.25×10^{-2}	1.46×10^{-3}	6.48×10^{-3}	↑
**28**	203.0927	Metabolite	L-Tryptophan	6.21×10^{-3}	2.27×10^{-2}	2.44×10^{-7}	6.94×10^{-6}	↑
**29**	207.1758	Metabolite	5-Tetradecenoic acid	4.94×10^{-3}	1.97×10^{-2}	2.09×10^{-2}	4.60×10^{-2}	↑
**30**	1745.82	Peptide	Pep_1745.82_UMOD	3.24×10^{-3}	1.59×10^{-2}	2.19×10^{-5}	2.37×10^{-4}	↓
**31**	1755.89	Peptide	Pep_1755.89_UMOD	1.82×10^{-5}	2.49×10^{-4}	1.02×10^{-2}	3.45×10^{-2}	↓
**32**	1790.74	Peptide	Pep_1790.74	1.49×10^{-3}	9.30×10^{-3}	9.35×10^{-6}	1.13×10^{-4}	↓
**33**	1894.33	Peptide	Pep_1894.33_UMOD	5.17×10^{-5}	6.27×10^{-4}	4.72×10^{-8}	1.62×10^{-6}	↓
**34**	1912.08	Peptide	Pep_1912.08_UMOD	2.21×10^{-8}	6.49×10^{-7}	2.22×10^{-8}	9.15×10^{-7}	↓
**35**	1938.08	Peptide	Pep_1938.08_UMOD	6.75×10^{-3}	2.78×10^{-2}	2.40×10^{-4}	1.50×10^{-3}	↓
**36**	1950.25	Peptide	Pep_1950.25_UMOD	3.42×10^{-3}	1.64×10^{-2}	2.57×10^{-5}	2.65×10^{-4}	↓
**37**	2040.25	Peptide	Pep_2040.25_UMOD	8.17×10^{-5}	8.85×10^{-4}	6.10×10^{-5}	5.23×10^{-4}	↓
**38**	2059.03	Peptide	Pep_2059.03_SERPINA1	6.44×10^{-4}	4.91×10^{-3}	4.75×10^{-4}	2.72×10^{-3}	↑
**39**	2176.33	Peptide	Pep_2176.33_VDAC1	9.45×10^{-4}	6.49×10^{-3}	2.17×10^{-12}	4.47×10^{-10}	↑
**40**	2336.89	Peptide	Pep_2336.89_SERPINC1	2.97×10^{-6}	5.55×10^{-5}	1.45×10^{-6}	2.71×10^{-5}	↑
**41**	2386.21	Peptide	Pep_2386.21_AMBP	4.44×10^{-9}	1.52×10^{-7}	4.47×10^{-5}	4.19×10^{-4}	↑
**42**	2520.43	Peptide	Pep_2520.43_SERPINA1	1.08×10^{-13}	1.11×10^{-11}	2.59×10^{-10}	1.78×10^{-8}	↑
**43**	2527.42	Peptide	Pep_2527.42_VDAC2	5.52×10^{-10}	2.27×10^{-8}	1.82×10^{-6}	6.17×10^{-8}	↑
**44**	2712.31	Peptide	Pep_2712.31_IGF2	1.34×10^{-2}	4.68×10^{-2}	8.33×10^{-3}	2.96×10^{-2}	↑

Table S5. LC-MS/MS identification of potential metabolite peaks for discrimination of patients with diabetic kidney disease.

No.	Measured *m/z*	Theoretical *m/z*	Adductive ions	Identification	Delta (ppm)	Main fragments	Database
**1**	102.0587	102.0561	M-H	GABA	26	58,84	HMDB
**2**	103.0045	103.0037	M-H	Malonic acid	8	41,59,85	HMDB
**3**	104.0361	104.0353	M-H	Serine	8	43,56,74	HMDB
**4**	110.0277	110.0270	M+H	Hypotaurine	6	67,81,83,93	HMDB
**5**	109.0327	109.0295	M-H	Hydroquinone	29	53,65,81	HMDB
**6**	112.0517	112.0505	M+H	Cytosine	10	52,67,68,69,95	HMDB
**7**	111.0229	111.0200	M-H	Uracil	26	42,68	HMDB
**8**	116.0713	116.0706	M+H	L-Proline	6	53,55,70,71,98	HMDB
**9**	118.0856	118.0863	M+H	L-Valine	6	56,57,58,60,72	HMDB
**10**	120.0667	120.0655	M+H	L-Threonine	10	56,74,102	HMDB
**11**	122.0279	122.0270	M+H	L-Cysteine	7	61,76	HMDB
**12**	122.0256	122.0248	M-H	Nicotinic acid	7	78,95	HMDB
**13**	126.0247	126.0219	M+H	Taurine	22	73,80,90	HMDB
**14**	125.0273	125.0244	M-H	5-Methylfuran-2-carboxylic acid	23	63,81,89,107	HMDB
**15**	128.0374	128.0353	M-H	L-Pyroglutamic acid	16	52,59,72,82	HMDB
**16**	130.0514	130.0510	M-H	N-Acetylalanine	3	70,82,88,114	HMDB
**17**	134.0463	134.0448	M+H	L-Aspartic acid	11	77,79,89,116	HMDB
**18**	134.0485	134.0472	M-H	Adenine	10	41,53,66,80,92,105,107,117	HMDB
**19**	137.0264	137.0244	M-H	Salicylic acid	14	65,67,93,109	HMDB

**20**	139.0518	139.0513	M-H	Imidazolepropionic acid	4	67,95,121	HMDB
**21**	142.0838	142.0874	M-H	Proline betaine	25	45,53,59,69,83,85	HMDB
**22**	144.0662	144.0666	M-H	Allysine	3	54,55,58,72,82,100,102,126	HMDB
**23**	148.0424	148.0438	M-H	L-Methionine	9	47,73,86,100,115	HMDB
**24**	149.0220	149.0244	M-H	Phenylglyoxylic acid	16	77,89,103,105	HMDB
**25**	151.0277	151.0261	M-H	Xanthine	10	65,81,108,124,134	HMDB
**26**	154.0490	154.0499	M+H	3-Hydroxyanthranilic acid	6	53,80,92,108,136	HMDB
**27**	153.0219	153.0193	M-H	Gentisic acid	17	53,69,81,109	HMDB
**28**	154.0638	154.0622	M-H	L-Histidine	10	67,72,80,81,93,110,137	HMDB
**29**	155.0471	155.0462	M-H	Imidazolelactic acid	6	67,81,111	HMDB
**30**	156.0686	156.0666	M-H	N-Acetylproline	13	83,112	HMDB
**31**	165.0218	165.0193	M-H	Phthalic acid	15	77,93,103,121	HMDB
**32**	167.9970	167.9972	M-H	Cysteic acid	1	72,77,81,88,95,107,124,150	HMDB
**33**	170.0442	170.0453	$M-H_2O-H$	N-Acetyl-L-glutamic acid	7	58,72,85,100,114,128,144,146	HMDB
**34**	173.1041	173.1044	M-H	L-Arginine	2	112,131,155	HMDB
**35**	180.0679	180.0666	M-H	L-Tyrosine	7	72,93,119	HMDB
**36**	190.0522	190.0543	M-H	N-Acetylmethionine	11	116,128,131,144,146,172	HMDB
**37**	203.0842	203.0826	M-H	L-Tryptophan	8	72,74,116,130,142,159	HMDB
**38**	207.1727	207.1749	$M-H_2O-H$	5-Tetradecenoic acid	11	59,71,97,109,125,135,139,149,163	HMDB

Table S6. MALDI-TOF/TOF tandem mass spectrometry identification of urine metabolites with commercial standard reagents.

No.	Identification	Detected m/z in standard by MALDI-MS	MS/MS fragments of standard in MALDI	Detected m/z of urine by MALDI-MS	MS/MS fragments of urine metabolites in MALDI	Possible metabolites	Molecular formula	Theoretical m/z
**1**	standard*	102.0563	42,58,71,84	102.0608	58,71,84	GABA	$C_4H_9NO_2$	102.0561
**2**	standard	103.0065	41,44,59,72,75,85	102.9920	41,44,59,72,75,85	Malonic acid	$C_3H_4O_4$	103.0037
**3**	standard*	104.0350	58,60,74	104.0349	58,60,74	Serine	$C_3H_7NO_3$	104.0353
**4**	standard*	108.0125	64,65	108.0157	64,65	Hypotaurine	$C_2H_7NO_2S$	108.0125
**5**	standard*	109.0302	41, 65,79,81,83	109.0280	41,65,79,81,83	Hydroquinone	$C_6H_6O_2$	109.0295
**6**	standard*	110.0363	42,67,84	110.0353	42,67,84	Cytosine	$C_4H_5N_3O$	110.0360
**7**	standard*	111.0203	42,68,84,85	111.0217	42,68,84,85,93	Uracil	$C_4H_4N_2O_2$	111.0200
**8**	standard*	114.0566	45,68,86	114.0530	45,68,86	L-Proline	$C_5H_9NO_2$	114.0561
**9**	standard	116.0715	45,70,75,89,93,99	116.0686	45,70,75,89,93	L-Valine	$C_5H_{11}NO_2$	116.0717
**10**	standard*	118.0498	56,72,74	118.0534	56,72,74	L-Threonine	$C_4H_9NO_3$	118.0510
**11**	standard	120.0115	76,93,100	120.0128	76,93,100	L-Cysteine	$C_3H_7NO_2S$	120.0125
**12**	Standard	122.0251	45,77,78,95,100,105	122.0261	77,78,95,100,105	Nicotinic acid	$C_6H_5NO_2$	122.0248
**13**	standard*	124.0071	60,65,79,80,95,105,107	124.0010	60,65,79,80,95,105	Taurine	$C_2H_7NO_3S$	124.0074
**14**	standard*	125.0275	41,81,107	125.0277	41,81,107	5-Methylfuran-2-carboxylic acid	$C_6H_6O_3$	125.0244
**15**	standard	128.0343	82,84,102,103,110	128.0347	71,82,84,102,103,110	L-Pyroglutamic acid	$C_5H_7NO_3$	128.0353
**16**	standard*	130.0515	42,70,88,112	130.0465	42,70,88,112	N-Acetylalanine	$C_5H_9NO_3$	130.0510
**17**	standard*	132.0305	42,71,72,88,115	132.0221	42,71,72,88	L-Aspartic acid	$C_4H_7NO_4$	132.0302
**18**	standard*	134.0473	78,92,105,107,117	134.0551	78,92,107,117	Adenine	$C_5H_5N_5$	134.0472

**19**	standard*	137.0244	77,93,119	137.0246	41,77,93,119	Salicylic acid	$C_7H_6O_3$	137.0244
**20**	standard*	139.0516	67,94,121	139.0515	41,67,94,112,121	Imidazolepropionic acid	$C_6H_8N_2O_2$	139.0513
**21**	standard*	142.0875	96,99,115,121	142.0704	83,85,96,99,115,121	Proline betaine	$C_7H_{13}NO_2$	142.0874
**22**	standard*	144.0621	41,100,116,126	144.0626	41,100,108,116,126	Allysine	$C_6H_{11}NO_3$	144.0666
**23**	standard	148.0439	47,100,123	148.0404	47,100,123	L-Methionine	$C_5H_{11}NO_2S$	148.0438
**24**	standard*	149.0243	63,77,105,123	149.0332	63,105,123	Phenylglyoxylic acid	$C_8H_6O_3$	149.0244
**25**	standard*	151.0257	42,81,108,124,133	151.0328	108,124,133	Xanthine	$C_5H_4N_4O_2$	151.0261
**26**	standard*	152.0356	78,108,126,134	152.0288	78,92,108,126,134	3-Hydroxyanthranilic acid	$C_7H_7NO_3$	152.0353
**27**	standard*	153.0188	41,81,107,109,123,125,135	153.0201	41,107,109,123,125	Gentisic acid	$C_7H_6O_4$	153.0193
**28**	standard*	154.0626	72,81,93,109,110,137	154.0590	93,109,110,137	L-Histidine	$C_6H_9N_3O_2$	154.0622
**29**	standard*	155.0463	40,67,81	155.0451	40,67,81	Imidazolelactic acid	$C_6H_8N_2O_3$	155.0462
**30**	standard*	156.0657	43,58,96,114,138	156.0677	43,96,114,138	N-Acetylproline	$C_7H_{11}NO_3$	156.0666
**31**	standard*	165.0191	77,121,147	165.0147	77,103,121,147	Phthalic acid	$C_8H_6O_4$	165.0193
**32**	standard*	167.9969	81,86,124,150,151	167.9989	86,124,150,151	Cysteic acid	$C_3H_7NO_5S$	167.9972
**33**	standard*	170.0430	59,100,128,144	170.0331	59,100,128,144	N-Acetyl-L-glutamic acid	$C_7H_{11}NO_5$	170.0453
**34**	standard*	173.1052	114,131,156	173.1044	99,114,131,156	L-Arginine	$C_6H_{14}N_4O_2$	173.1044
**35**	standard*	180.0650	72,93,119,139,163	180.0696	72,93,119,139	L-Tyrosine	$C_9H_{11}NO_3$	180.0666
**36**	standard	190.0549	115,47,129,142,148	190.0619	47,129,142,148	N-Acetylmethionine	$C_7H_{13}NO_3S$	190.0543
**37**	standard*	203.0818	74,116,142,159,174	203.0927	74,142,159,174	L-Tryptophan	$C_{11}H_{12}N_2O_2$	203.0826
**38**	standard	207.1738	111,125,135,139,149,163	207.1758	111,125,135,149,163	5-Tetradecenoic acid	$C_{14}H_{26}O_2$	207.1749

Note: Because the ion source of two MS technologies (UPLC-MS and MALDI-TOF MS) are different, so the tandem MS results are not comparable, * represents the MALDI -TOF/TOF tandem mass spectrometry results of some metabolites are consistent with the UPLC-MS/MS data.

Table S7. Nano-LC-ESI MS/MS identification of urine peptides.

Measured m/z	Theoretical m/z	Amino sequence	Delta (ppm)	Modification	Protein name
960.637	960.56	FQNAILVR	80.16157		
1047.361	1047.51	DFSFLPQPP	-142.242		COL1A1
1086.569	1086.60	MFSLKPPKP	-28.5294	0, Acetyl[AnyN-term]	MTMRA
1103.988	1103.69	LPGVPLARPAL	88.79305		OAF
1149.796	1149.62	LVQEVTDFAK	153.0941		ALB
1615.784	1615.89	NELRVAPEEHPVLL	-65.5985		
1745.816	1745.90	IDQSRVLNLGPITR	-48.1127	3, Xlink_DFDNB[Q]	UMOD
1755.890	1755.96	SGSVIDQSRVLNLGPIT	-39.8642		UMOD
1790.735	1790.89	SYELPDGQVITIGNER	-86.5491		
1894.326	1894.05	SGSVIDQSRVLNLGPITR	145.7195	3, Dehydrated[S]	UMOD
1912.077	1912.07	SGSVIDQSRVLNLGPITR	3.660954		UMOD
1938.077	1937.96	DQSRVLNLGPITR	60.37276	3, Hex(1)NeuGc(1)[S]	UMOD
1950.251	1949.96	IIVDTYGGWGAHGGGAFSGK	149.2338		UMOD
1973.138	1972.94	IQKLESDVSAQMEYCR	100.3578	12, Oxidation[M];<br>15, Carbamidomethyl[C]	FGB
2040.252	2040.16	SGSVIDQSRVLNLGPITRK	45.0945		UMOD
2059.025	2059.09	EAIPMSIPPEVKFNKPFV	-31.5673	5, Oxidation[M]	SERPINA1

2176.330	2176.06	WNTDNTLGTEITVEDQLAR	124.0775		VDAC1
2336.893	2337.17	AFLEVNEEGSEAAASTVISIAGR	-118.519	21, Ala->Ser[A]	SERPINC1
2386.211	2386.15	MTVSTLVLGEGATEAEISMTSTR	25.56419	7, Val->Thr[V]	AMBP
2437.243	2437.19	ERDVSTPPTVLPDNFPRYPVG	21.74636	2, Arg->Npo[R]	IGF2
2451.026	2451.19	LLGDASVSFTENCVVGIQANTER	-66.9063	10, Formyl[T](Thr->Glu[T])	FH
2520.430	2520.34	LMIEQNTKSPLFMGKVVNPTQK	35.70947	2, Oxidation[M]; 6, Deamidated[N]	SERPINA1
2527.419	2527.19	TGDFQLHTNVNDGTEFGGSIYQK	90.61448	12, Asp->Asn[D]	VDAC2
2712.306	2712.35	ERDVSTPPTVLPDNFPRYPVGKF	-16.2221	2, Arg->Npo[R]	IGF2
